# Electromyographic responses of the auriculomotor system during effortful listening in hearing aid users

**DOI:** 10.3389/fnins.2026.1838748

**Published:** 2026-08-04

**Authors:** Andreas Schroeer, Tanja Biehl, Matthias Latzel, Stefan Launer, Daniel J. Strauss, Farah I. Corona-Strauss

**Affiliations:** 1 Systems Neuroscience and Neurotechnology Unit, Faculty of Medicine, Saarland University & htw saar, Homburg, Saar, Germany; 2 Saarland University, Faculty of Medicine, Homburg, Saar, Germany; 3 Center for Digital Neurotechnologies Saar, Homburg, Saar, Germany; 4 Auris Hörcenter GmbH, St. Wendel, Germany; 5 Sonova AG, Stäfa, Switzerland

**Keywords:** auricular muscles, effortful listening, EMG (electromyography), hearing aids, noise reduction, objective measures, posterior auricular muscle, superior auricular muscle

## Abstract

**Background:**

Auricular muscles in humans may still display increased activity associated with auditory attention and effortful listening in complex patterns. As research into this auriculomotor system has primarily been conducted in normal-hearing participants, this study investigated electromyographic (EMG) signals of auricular muscles in hearing aid users under challenging listening conditions in order to evaluate their potential future use as a measure of perceived listening effort.

**Methods:**

Twenty-four experienced, bilateral hearing aid users (mean age approximately 64 years) were instructed to attend a target podcast, which was either presented in quiet (SIQ), or in front of a spatially distributed, noisy background. If noise was present, a specialized noise reduction feature of the hearing aids was either turned off (NR-off) or on (NR-on), which resulted in three task difficulties, or conditions: SIQ, NR-on, and NR-off. EMGs of two auricular muscles, the superior and posterior auricular muscles (SAM, PAM), as well as the corrugator supercilii (part of the facial muscles) were recorded.

**Results:**

Perceived listening effort significantly increased from SIQ to NR-on to NR-off, indicating that these three conditions presented distinct task difficulties. PAM activity resembled the perceived listening effort (highest activity during NR-off, intermediate during NR-on, lowest during SIQ), but statistical significance was only observed between NR-off and SIQ (*p* = 0.013). Activity of the corrugator significantly increased over time in the presence of noise, regardless of the noise reduction algorithm, but remained low during SIQ. While we did not observe a significant effect of task difficulty on the SAM, we unexpectedly found a strong time-dependent decrease in SAM activity, which likely poses as a serious confounding factor.

**Conclusions:**

This preliminary study shows that, in a group of hearing aid users, the activity of auricular muscles increased in highly demanding listening tasks, but due to an unexpected time drift, replication of the study with a modified protocol and a larger participant group is necessary.

## 1 Introduction

Humans still posses a total of nine auricular muscles per ear (Standring, 2016). However, their overt function to reorient the pinna toward a particular sound of interest (as commonly observed in, for example, cats or dogs) has been reduced to a vestigial state over the last 25 million years (Hackley, 2015). Nevertheless, a very fast, reflexive response to brief acoustic stimuli (e.g., clicks) can still be observed at the posterior auricular muscle (PAM) approximately 10–20 ms after stimulus onset (Kiang et al., 1963), and has been thoroughly investigated in the past, mostly in relation to objective hearing tests (Douek et al., 1974; O'Beirne and Patuzzi, 1999; Patuzzi and O'Beirne, 1999; Purdy et al., 2005) and emotional-affective modulation (Benning et al., 2004; Hess et al., 2007; Gable and Harmon-Jones, 2009; Hackley et al., 2009).

In addition to this fast reflex, more recent research by Strauss et al. (2020) has found that electromyographic (EMG) signals of several auricular muscles are dependent on the spatial position of acoustic signals, and may also be an indicator of a person's voluntary auditory attention. This “neural fossil” consists of a relatively fast (approximately 70 ms after stimulus onset), transient response, which involves multiple auricular muscles, is highly dependent on the direction of auditory stimuli, and can contain complex, antagonistic components, i.e., the activity of some auricular muscles may be suppressed, while the activity of others is simultaneously increased (Schroeer et al., 2023, 2024, 2025b). Additionally, Strauss et al. (2020) reported a sustained component which lateralizes auricular EMG activity when participants focused their attention toward a specific speaker in space, while ignoring a concurrent, but spatially separated distractor (i.e., a “cocktail-party-scenario,” see Cherry, 1953). Furthermore, these response types (transient and sustained) were generally larger when the auditory stimuli were presented from outside the participants field-of-view, compared to inside their field-of-view (a behavior which has also been observed by Olszanowski et al. (2023)). Overall, these studies provide evidence that the auriculomotor system in humans is still heavily affected by auditory attention.

Listening effort, which is similar to, but still distinct from, auditory attention (Kahneman, 1973; Bruya and Tang, 2018) can be described as “the deliberate allocation of mental resources to overcome obstacles in goal pursuit when carrying out ... [a listening task]” (Pichora-Fuller et al., 2016), and has been investigated using a large array of self-reported, behavioral, and physiological measures (Guijo and Cardoso, 2018; Alhanbali et al., 2019; Shields et al., 2023; Keur-Huizinga et al., 2024). As spatially separating the target from distractor speakers can significantly decrease the perceived listening effort (Rennies and Kidd, 2018; Rennies et al., 2019), especially with an increasing (angular) distance between target and distractor (Krueger et al., 2022), Schroeer et al. (2025a) conducted an experiment aimed at investigating responses of auricular muscles during effortful listening. The hypothesis was that under more effortful listening conditions, the auriculomotor system should increase in activity, as the reorienting or reshaping of the pinna could have once aided in stream segregation, and therefore lowering the perceived listening effort (for the effect of artificial/exaggerated changes of the pinna on sound localization see Oldfield and Parker, 1984; Trapeau et al., 2016; Shirota et al., 2019; Stevenson-Hoare et al., 2022). Schroeer et al. (2025a) found that in the most effortful condition, activity of the superior auricular muscle (SAM) was significantly increased (somewhat similar to the results of Mackersie and Cones, 2011, but recorded at the frontalis muscle). The advantages of using auricular muscles in listening effort research are twofold. First, as a tool in auditory research in general. As listening effort is a complex concept, different metrics have been shown to be sensitive to different dimensions of listening effort (Alhanbali et al., 2019). Therefore, the addition of auricular muscles could potentially help to understand certain aspects of listening effort. Second, from an applied point-of-view, electrodes are fairly easy to place at the auricular muscles, especially at the PAM, because it is located at a hairless area (at the mastoid process behind the ear). Additionally, signals from auricular muscles can also be recorded from in-ear electrodes (Schroeer et al., 2023). Overall, they can be easily and discreetly recorded, especially in the context of hearing aids (or hearables), as a technical system, including a power supply, is practically directly located at the auricular muscles. Therefore, signals obtained from auricular muscles could, potentially, in the future be utilized in closed feedback loops in hearing aid or hearable users. Nevertheless, this effect was only observed approximately two minutes into the listening task, as differences between the most effortful condition and the least/intermediate conditions increased over time. Activity of the posterior auricular muscle (PAM), which was previously found to be very active during spatial auditory attention tasks, remained unexpectedly unaffected.

While Schroeer et al. (2025a) found that some aspects of the auriculomotor system were affected by effortful listening, they did so in a group of relatively young (≈28 years old), normal-hearing participants. A relatively straightforward follow-up to Schroeer et al. (2025a) is therefore if similar results could be obtained in a group of hearing aid users. There is a large body of research detailing the effects of hearing aid usage on listening effort (Downs, 1982; Picou et al., 2013; Vaisberg et al., 2024), as well as the potential benefits of features such as directional microphones and noise reduction algorithms. For example, specialized noise reduction algorithms have been demonstrated to reduce listening effort, as measured by self-reported questionnaires (Desjardins and Doherty, 2014), response times (Reinten et al., 2021), pupil dilation (Fiedler et al., 2021), or functional near infrared spectroscopy (Vaisberg et al., 2025). Similar benefits have been demonstrated when using directional microphones/beamformers, quantified similarly by self-reported, behavioral, and physiological markers (Bernarding et al., 2017; Picou and Ricketts, 2018; Bernarding et al., 2017). However, it should also be mentioned that comparisons between studies can be very challenging, as most studies were conducted with very different paradigms, participant demographics, and outcome measures, as discussed in a systematic review by Ohlenforst et al. (2017). Interestingly, Ohlenforst et al. (2017) also identified a few studies in which hearing aid amplification resulted in an increased listening effort, highlighting the variability of such studies.

Nevertheless, the current study aimed to assess the effects of effortful listening on the auriculomotor system in a more applied setting by (a) recruiting hearing aid users, (b) performing measurements at a hearing aid acoustician store, and (c) using the hearing aid's noise reduction algorithm to reduce the perceived listening effort in one condition. While the outcome measures (EMGs of the PAM and SAM) are identical to those in Schroeer et al. (2025a), we will utilize spatially distributed noise sources (similar to a “cocktail party scenario,” as mentioned earlier) instead of co-locating target and noise streams. This change does not only make for a more realistic, ecologically valid scenario, but the utilized noise reduction algorithm is much more effective in such a scenario. While Schroeer et al. (2025a) has only reported a significant effect of effortful listening on the SAM, but not the PAM, their experiment did not have any spatially distributed sound sources. As the PAM has been shown to be highly influenced by auditory attention and the spatial direction of sound sources (Strauss et al., 2020; Schroeer et al., 2023, 2025b), the PAM may show some activity in the scenario of the current study. Therefore, our main research hypothesis is that the reduction of the spatially distributed noise (due to the noise reduction algorithm) should reduce the perceived listening effort, which should in turn be reflected by a reduction or change in SAM and PAM activity.

## 2 Material and methods

### 2.1 Participants

**Figure 1:**
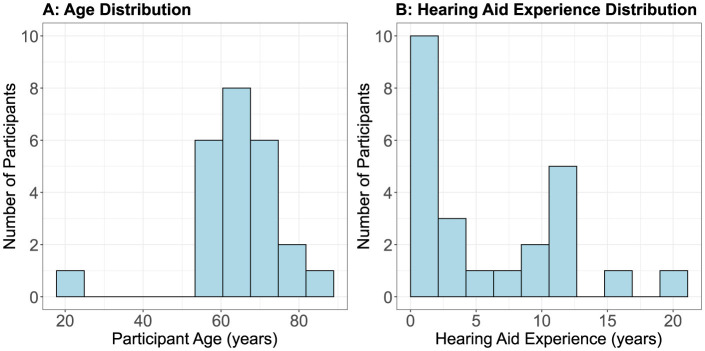
Histograms of participant age and hearing aid experience. **(A)** Age distribution. **(B)** Hearing aid experience distribution.

Participants were bilateral hearing aid users with symmetric, slight to severe hearing impairment (according to the WHO's grading scheme, see Olusanya et al., 2019), recruited from the customer database of a local hearing aid acoustician store (Auris Hörcenter GmbH located in St. Wendel, Saarland, Germany). All participants were furthermore required to have at least one year of experience with hearing aids. Initially, 30 participants were recruited, but six participants were excluded because they (a) displayed a noticeable tremor during the experiment, (b) fell asleep, (c) reported to suffer from tinnitus, and (d) displayed extreme difficulty understanding speech in noise. Of the 24 included participants (15 male, 9 female), one participant was 18 years old, while the remaining were between 54 and 82 years old (66.17 ± 7.70 years). Participants had, on average, 6 years of hearing aid experience, see Figure 1 for more details.

**Figure 2:**
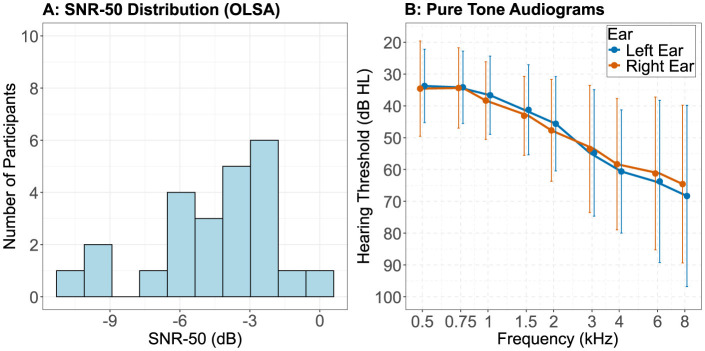
**(A)** Histogram of SNR-50 values of the OLSA sentence matrix test. Speech-shaped noise was kept at a constant 60 dB, while the intensity of test sentences were adapted until 50% were correctly recalled. This test was conducted using the hearing aids described in 2.3, with the noise reduction algorithm turned off. **(B)** Averages and standard deviations of the (unaided) pure tone audiograms of all included participants.

Furthermore, the participant's ability to understand speech in noise was assessed by a German sentence matrix text (Oldenburger Satztest, Wagener et al., 1999), during which participants wore the hearing aids used for this study (see 2.3 for more details). The results are displayed in Figure 2A. Pure tone audiograms were performed using test frequencies of 500, 1,000, 2,000, and 4,000 Hz. Averaged results were 44.17 dB HL on the left, and 44.74 dB HL on the right ear (see Figure 2B for the full averaged audiogram).

All participants gave written, informed consent after the experiment was explained to them in detail. For their participation in the study, participants received a 35€gift card. The study was approved by the responsible ethics committee (ethics commission at the Ärztekammer des Saarlandes, Saarbrücken, Germany; Identification Number: 02/25).

### 2.2 Experimental setup

**Figure 3:**
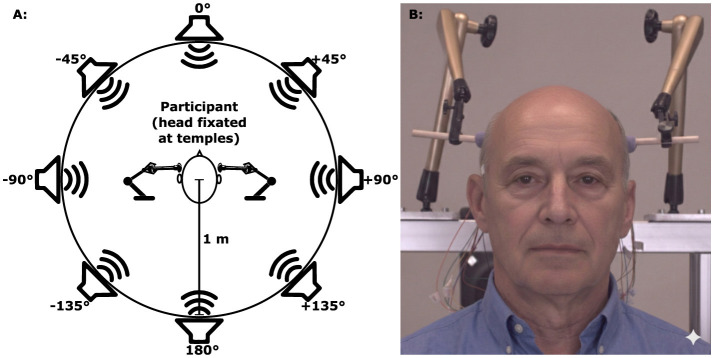
**(A)** Schematic of the experimental setup, visualizing the positions of the loudspeakers relative to the participant. **(B)** Exemplary image of one participant with fixations at the temples to assist in preventing head movements. The face of the participant was generated, and the shirt was altered by generative AI (Gemini 2.5 Flash Image).

Participants were seated in a chair, in a hearing aid fitting room at the Auris Hörcenter GmbH. In a circle around them, eight active loudspeakers (KH120A, Neumann, Germany) were positioned 1 meter away from the participant, at head level, in steps of 45°. Below the loudspeaker which participants were facing (at 0°), a fixation cross was placed and participants were instructed to fixate their gaze on this, and to avoid eye and head movements as good as possible during the experiment. In order to further support the participants in minimizing head movements (in particular tilting or rotations to the side), two magic arms were attached to a metal frame placed behind the backrest of the chair. At the end of these magic arms, soft foam tips were attached. These foam tips were gently placed at the left and right temporal regions of the participants in order to stabilize their heads. A schematic of the setup is displayed in Figure 3. Audio stimulation was controlled by a dedicated PC, via an external USB sound interface (Scarlett 18i20, Focusrite Plc., UK), which generated audio and trigger signals at a sampling frequency of 44.1 kHz. Additionally, this PC controlled the mode of the hearing aids used in the experiment (noise reduction algorithm turned on/off), which is described in 2.3.

### 2.3 Hearing aids and noise reduction algorithm

For the experiment, participants were provided with a pair of Phonak Audeo I90-SPHERE hearing aids with SlimTips, closed vents, and M receivers. Hearing aids were fitted using the Adaptive Phonak Digital 3.0 (APD 3.0) fitting formula, with a gain of 100%. Feedback reduction was activated, and frequency lowering was deactivated. The microphone mode was set to “real ear sound,” which simulates the directional characteristics of the ear.

At specific points during the experiment, an AI-based noise reduction algorithm, which is implemented on a neural network processing chip in the hearing aids, was activated. This noise reduction algorithm, called “Spheric Speech Clarity,” is based on a deep neural network with 4.5 million parameters and was specifically trained on 22 million sound samples to separate speech from noise in a “cocktail party scenario,” and operates on each hearing aid independently. The algorithm calculates the complex input spectrum of 64 frequencies. Based on this, the DNN computes and applies a 64-frequency mask, which is designed to separate noise from the speech audio signal. Furthermore, according to the manufacturer, the algorithm can increase the SNR by up to 10 dB under optimal conditions. It should also be noted that in the implementation used in this study, after the signal components which were identified as noise were removed, the remaining (speech) signal is additionally amplified. For more technical details about the algorithm, see Hasemann and Krylova (2024).

### 2.4 Stimuli and tasks

Segments from an audiobook, spoken by a female, dialect-free german speaker discussing a variety of topics were used (the same as the target stream in Schroeer et al., 2025a). Pauses during speech were removed by first calculating the moving average of a length equivalent to 100 ms on the rectified audio files. Then, all instances in which this smoothed waveform was below 0.001 (arbitrary units) were removed. These parameters (length of the moving average and threshold value) were chosen so no words of the processed audio file were cut off or contained unnaturally sounding transitions between words.

Additionally, spatially distributed, ecologically valid noise was presented from all eight loudspeakers.

During the experiment, participants were instructed to always attend the audiobook, while ignoring the competing, spatialized noise streams. The audiobook was always presented from the loudspeaker at 0°, facing the participant, and at an intensity of 58 dBA. The noise signals, if presented, were played from all eight loudspeakers at 51 dBA each, resulting in a total noise intensity of 60 dBA, and consequently an SNR of –2 dB.

The experiment consisted of three conditions (or blocks), with the presentation order counterbalanced across participants. It should be noted that, when not specifically stated otherwise, the noise reduction algorithm described in 2.3 was turned off.

**Figure 4:**
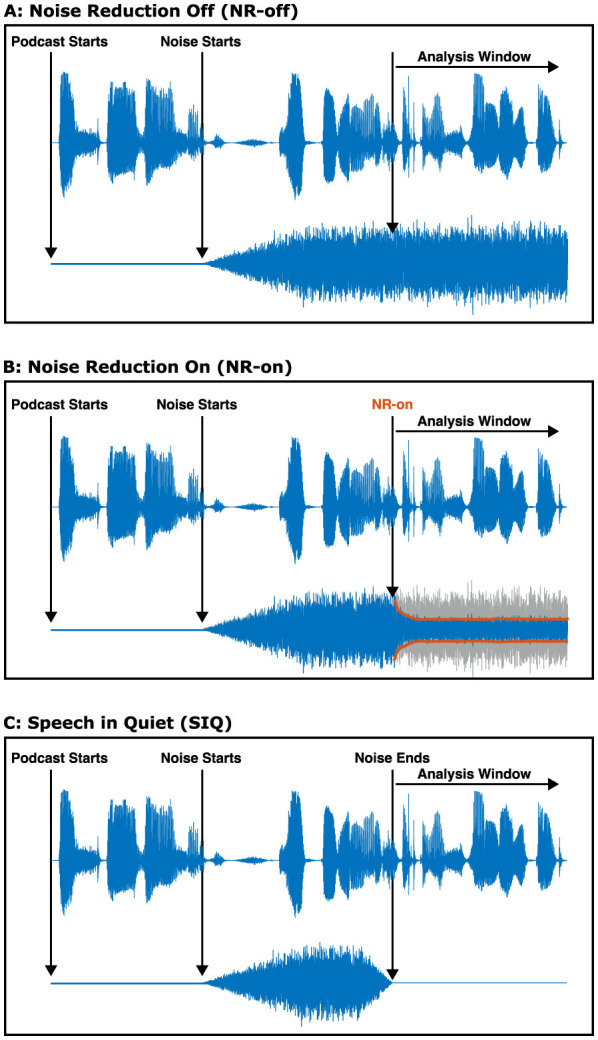
Visualization of the timing between target, distractor, and hearing aid mode for all three conditions. Note that the waveforms and distances along the axes are for simplified visualization purposes only and not to scale. **(A)** Noise reduction off (NR-off). **(B)** Noise reduction on (NR-on). **(C)** Speech in quiet (SIQ).

In the first condition, referred to as Noise Reduction Off (NR-off, see Figure 4), the audio stimulation started by presenting the podcast without any noise for 20 seconds. During this clean speech segment, participants were able to get used to and focus on the target podcast. After 20 seconds of presenting the podcast only, noise was linearly faded in, and reached its maximum intensity after 10 seconds. Participants were given another seven additional seconds, during which they could get acclimated to the final SNR. After this, the podcast and noise was presented for 270 seconds, which was considered the analysis window for the collected EMG data.

The second condition, the Noise Reduction On condition (NR-on, see Figure 4B), was almost identical to the NR-off condition. The only difference was that five seconds after the noise reached maximum intensity, the noise reduction algorithm mentioned in 2.3 was activated, and the analysis window started two seconds after it was turned on. At the end of this condition's analysis window, the noise reduction algorithm was turned off again.

During the third condition, the target podcast was presented in quiet, which is why this condition is referred to as Speech in Quiet (SIQ). However, noise was still linearly faded in, and was held at 60 dB for five seconds, before quickly fading out again, which then marked the beginning of the analysis window, see Figure 4C.

The motivation behind this was to make the difference between conditions less obvious to the participants, especially the difference between NR-on and SIQ. While the order of the conditions, as mentioned before, was counterbalanced, all three conditions were presented in direct succession of each other, without any break in between, i.e., the end of one condition marked the beginning of the clean speech segment of the next condition. This resulted in a total runtime of approximately 15.5 minutes. However, this also means that there were no rest periods between conditions, as the target stream was constantly playing from the frontal loudspeaker. Afterward, participants were asked to rate their perceived listening effort (LE; from “effortless” to “extreme” on a 7-point scale), and how often they lost the target stream on a visual scale with the extremes being “rarely” and “often”, and “sometimes” in the center of the scale, with numerical values ranging from 1 to 10, referred to as “distractibility.”

### 2.5 Data acquisition

Surface EMG signals were recorded using passive Ag/AgCl electrodes and a commercially available biosignal amplifier (g.USBamp, g.tec, Austria) at a sampling frequency of 4.8 kHz. Electrodes were located, in pairs, at the left and right PAM, SAM, and corrugator supercilii muscles, with the ground electrode placed at the upper forehead. For the PAM and SAM, one electrode was placed where the PAM/SAM attaches to the pinna, while another electrode (which was used to calculate bipolar EMG signals) was placed approximately 1 cm away along the corresponding auricular muscle, which is identical to the positions used in Strauss et al. (2020) and Schroeer et al. (2025a). For the corrugator muscles, one electrode was placed at the medial end of the superciliary arch and the corresponding reference electrode approximately 1 cm away on the belly of the corrugator muscle (similar to the position in Van Boxtel and Jessurun, 1993). Prior to the application of the electrodes, the skin of the participants was prepared by wiping the area with a disinfectant, applying an abrasive skin preparation gel with a cotton swap, and then using conductive electrode cream to lower the impedances. Even though this procedure was carefully applied, as many participants had sensitive skin, average impedances at the PAM were 23 ± 20 KΩ, 31 ± 24 KΩ at the SAM, and 63 ± 34 KΩ at the corrugator. However, it should also be noted that the EMG is very robust for impedances below 55 KΩ (Hewson et al., 2003). In addition to the EMG data, the biosignal amplifier recorded the trigger signals mentioned in 2.2 in parallel, in order to synchronize EMG data with the audio stimulation and changes in hearing aid modes.

### 2.6 Signal processing

Signal processing and statistical analyses were performed using Matlab 2020a (Mathworks, USA), R 4.4.0 (R Core Team, 2024), and JASP 0.19.3 (JASP Team, 2025). Signals of the left and right PAM, SAM, and corrugator muscles were re-referenced to obtain bipolar EMG signals. EMG signals were then zero-phase IIR bandpass filtered between 10–500 Hz and a zero-phase 50 Hz comb filter was applied.

Signals were segmented into 1 second long, non-overlapping segments, and the energy of each one was calculated. Independently for each muscle and recording side, potentially movement-related artifacts were rejected by removing segments exceeding three standard deviations from the corresponding mean, similar to the procedure described in Strauss et al. (2020); Schroeer et al. (2025a). On Average, at the SAM, PAM, and corrugator 1.204%, 0.809%, and 0.862% of all segments were removed. As both Strauss et al. (2020) and Schroeer et al. (2025a) have observed time-dependent drifts in auricular muscles activity (generally falling in Strauss et al., 2020 and partially rising in Schroeer et al., 2025a), we furthermore segmented the signals into three equally long parts (90 seconds each). These were then averaged within each participant, muscle, and recording side. Next, they were normalized by dividing them by the averaged baseline activity, which were defined as the clean speech segments (between the beginning of the podcast and the onset of the noise, see Figure 4), which occurred before/after each condition. Lastly, data from the left and right muscles were averaged and subjected to statistical analysis with two factors which had three levels each: condition (NR-off, NR-on, and SIQ), and time (first, second, third 90 seconds segment) by linear mixed models. However, as the baseline normalized data were strictly positive and heavily right-skewed, we applied the Box Cox transform in order to obtain more normally distributed residuals with equal variances across groups (Box and Cox, 1964):


$$Y^{\left(\text{λ}\right)}=\{\begin{array}{ll} \frac{y^{\text{λ}}-1}{\text{λ}} & \left(\text{λ}\neq 0\right)\\ log(y) & (\text{λ}=0), \end{array}$$ (1)

where *y* are the baseline normalized data of the SAM, PAM, or corrugator. The parameter λ was determined by the boxcox function of the MASS package (Venables and Ripley, 2002), initially applied on a simplified linear model (*muscle* ~*condition* *time). If the optimized λ was very small (below 0.1), the logarithmic transform was applied. Then, linear mixed-effect models were fitted on the transformed data with condition, time, and gender as fixed effects, participants as random intercept and condition as a random slope:


$$\begin{array}{r} \text{transformed data}\sim \text{condition}*\text{time}*\text{gender}+\\ \left(1+\text{condition}|\text{participant}\right). \end{array}$$

Main and interaction effects, as well as pairwise post-hoc t-tests were performed by the lmerTest and emmeans packages (Kuznetsova et al., 2017; Lenth and Piaskowski, 2023). Degrees of freedom were calculated using the Kenward-Roger method and *p*-values were Bonferroni-Holm corrected for multiple comparisons.

Regarding subjective data (perceived LE and distractibility), non-parametric Friedman tests were used to test for differences between conditions, as implemented in JASP. Likewise, Bonferroni-Holm corrected Conover tests were used to assess pairwise differences, if Friedman tests indicated significant differences between conditions. Critical alpha-values were set to α = 0.05 for all hypothesis tests.

## 3 Results

### 3.1 Subjective measures

**Figure 5:**
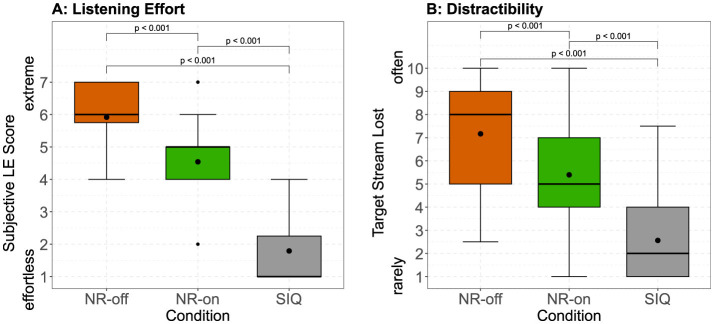
Boxplots of the subjective listening effort scores from 1 (effortless) to 7 (extreme), and distractibility ratings from 1 (rarely) to 10 (often). P-values were calculated using Bonferroni-Holm corrected Conover tests. **(A)** Listening effort. **(B)** Distractibility.

Results of the subjective LE scores and distractibility (how often participants lost the target stream) for all three conditions are displayed in Figure 5. Subjective LE was rated the highest in the NR-off condition (5.917), intermediate in the NR-on condition (4.542), and lowest during SIQ (1.792). A Friedman test revealed significant differences between groups ($\chi_{\left(2\right)}^{2}=38.719,p<0.001$). Furthermore, all pairwise comparisons were significantly different (all *p*-values < 0.001). Similar to the subjective LE scores, distractibility was also rated highest during NR-off, intermediate during NR-on, and lowest during SIQ (7.167, 5.396, 2.563). Differences between groups were statistically significant ($\chi_{\left(2\right)}^{2}=33.802,p<0.001$), and so were all pairwise comparisons (all *p*-values < 0.001).

**Figure 6:**
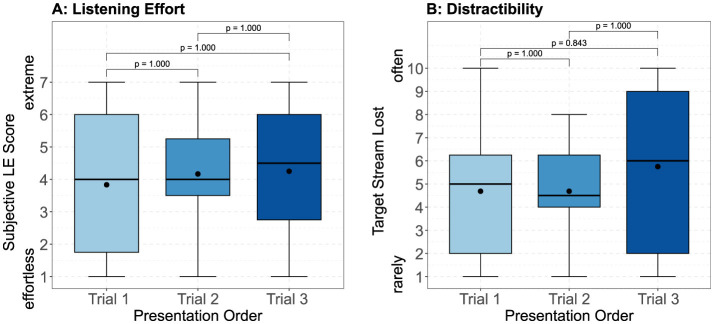
Boxplots of the subjective listening effort scores from 1 (effortless) to 7 (extreme), and distractibility ratings from 1 (rarely) to 10 (often), when re-arranged according to their presentation order. *P*-values were calculated using Bonferroni-Holm corrected Conover tests. **(A)** Listening effort. **(B)** Distractibility.

To assess potential confounding effects of the presentation order, data were re-arrange accordingly and analyses were run again, even though the average presentation order (1–3) for all three conditions were counterbalanced across participants. The average presentation order for each condition was: SIQ: 2.0, NR-off: 2.042, and NR-on: 1.958 (differences were not significant: $\chi_{\left(2\right)}^{2}=0.083,p=0.959$). For the subjective LE scores, no significant effect of presentation order was observed ($\chi_{\left(2\right)}^{2}=0.562,p=0.755$), even though the average score slightly increased (3.833, 4.167, 4.25). Similarly, the presentation order had no effect on the distractibility rating ($\chi_{\left(2\right)}^{2}=1.21,p=0.546$), with averaged ratings being 4.688, 4.688, 5.75, see Figure 6.

### 3.2 Electromyographic measures

**Figure 7:**
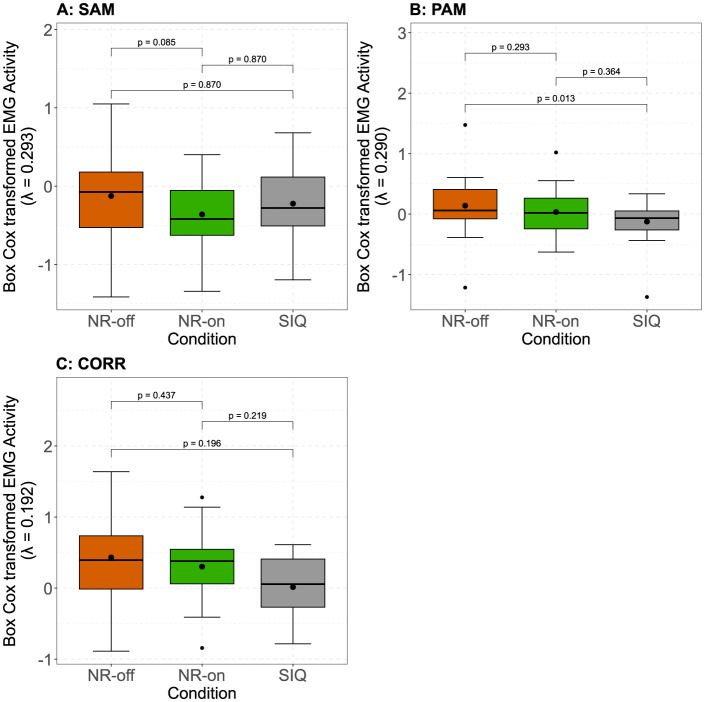
Boxplots of the averaged, normalized, Box Cox transformed activity of the SAM, PAM, and corrugator muscles in all three recorded conditions. The λ values in the y-axes indicate the parameter used in the Box Cox transform in Equation 1. *P*-values were calculated using Bonferroni-Holm corrected *t*-tests. **(A)** SAM. **(B)** PAM. **(C)** CORR.

Results of the SAM, PAM, and corrugator muscles, averaged according to the condition (NR-off, NR-on, SIQ) are presented in Figure 7 (averaged values prior to the Box Cox transformation can be seen in Supplementary Figure 1).

Data from the SAM displayed a different pattern than the subjective measures. Regarding averaged values, SAM activity, transformed back to the original scale, was highest during NR-off (0.916), intermediate during SIQ (0.789), and lowest during NR-on (0.711). It is notable that all of those values are below 1, which would correspond to the averaged baseline level. However, we did not observe a significant effect of condition [*F*_(2, 22)_ = 3.107, *p* = 0.065, see Figure 7A], time, gender, or any interaction between these factors (all *p*-values >0.1522).

The PAM, on the other hand, appeared to follow the subjective ratings more closely. PAM activity was, transformed back to the original scale, largest during NR-off (1.168), intermediate during NR-on (0.998) and lowest during SIQ (0.896). As opposed to the SAM, the main effect of condition was significant [*F*_(2, 22)_ = 5.268, *p* = 0.014]. Paired comparisons revealed that PAM activity during NR-off was significantly larger than during SIQ [*t*_(22)_ = 3.396, *p* = 0.013, Cohen's d = 1.212], while activity during NR-on was not significantly different from either of them, as displayed in Figure 7B. No other factors or interactions reached the significance threshold (all *p*-values >0.109).

Data obtained from the corrugator muscles were, on average, largest during NR-off (1.459), followed by NR-on (1.293), and by far the smallest during SIQ (1.02), displayed in Figure 7C. However, we did not observe a significant main effect of condition, but instead a highly significant main effect of time [*F*_(2, 131.996)_ = 19.473, *p* < 0.001], as well as a significant main effect of gender [*F*_(1, 22)_ = 6.15, *p* = 0.021], indicating higher corrugator activity in the male participants, and an interaction between time and condition [*F*_(4, 131.996)_ = 3.07, *p* = 0.019]. Concerning the main effect of time, post-hoc tests revealed that corrugator activity significantly increased from the first to the second and third segment (all *p*-values < 0.001, also see Supplementary Figure 2). However, analysis of the interaction, when fixing the level of condition as a factor, indicated that a time-dependent increase of activity from the first to the second/third segment was only present during NR-off and NR-on (all *p*-values < 0.01). A significant difference between the second and third segment was only observed during NR-on (*p* = 0.013) (see Supplementary Figure 3). During SIQ, no differences between (time) segments were observed. On the other hand, when fixing the time factor, we observed no differences between conditions during the first or second segment. However, during the third segment, activity during NR-off and NR-on were both larger than activity during SIQ [SIQ - NR-off: *t*_(27.5)_ = −2.392, *p* = 0.048, Cohen's d = 1.779; SIQ - NR-on: *t*_(31.8)_ = −2.768, *p* = 0.02, Cohen's d = 1.627] (also see Supplementary Figure 4). Summarizing, we observed a strong time-dependent increase at the corrugator, but only during NR-off and NR-on, whereas activity during SIQ remained stable. This likely manifests itself as a significant increase from NR-off/on compared to SIQ during the third segment.

**Figure 8:**
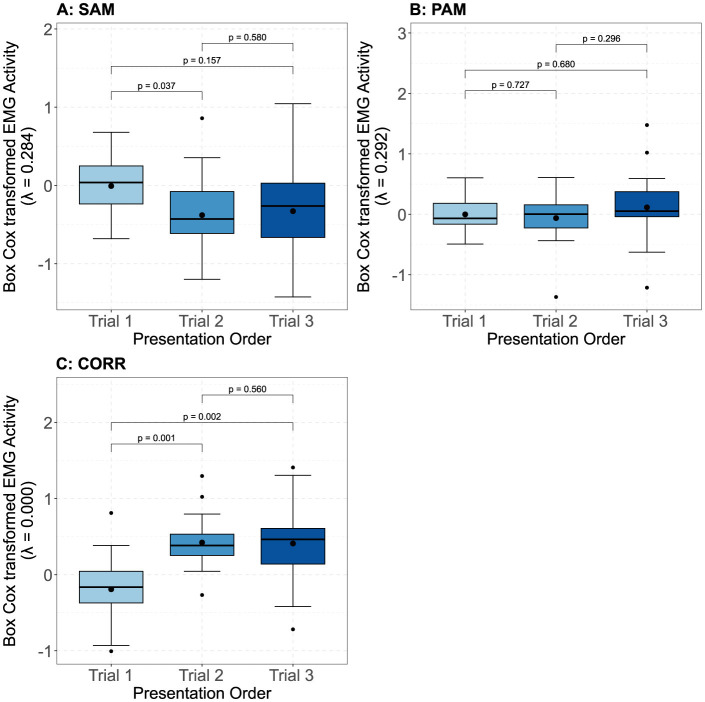
Boxplots of the averaged, normalized, Box Cox transformed activity of the SAM, PAM, and corrugator muscles when re-arranged according their presentation order. The λ values in the y-axes indicate the parameter used in the Box Cox transform in Equation 1. *P*-values were calculated using Bonferroni-Holm corrected *t*-tests. **(A)** SAM. **(B)** PAM. **(C)** CORR.

Data of the SAM, PAM, and corrugator muscles were also re-arranged according to the presentation order, see Figure 8 (also see Supplementary Figure 5 for data prior to applying the Box Cox transformation).

At the SAM (Figure 8A), we visually observed a drop of activity after the first trial (0.975, 0.692, 0.754 on the original scale). Note that during the first trial, the average value is practically 1, which corresponds to the averaged baseline, while the values during the second and third trial are much lower. We found a significant main effect of presentation order [*F*_(2, 22)_ = 4.103, *p* = 0.031], but also a significant interaction between presentation order and time [*F*_(4, 132)_ = 3.126, *p* = 0.017] and an interaction between all three factors: presentation order, time, and gender [*F*_(4, 132)_ = 3.438, *p* = 0.01]. The main effect of presentation order only suggests a significant decrease of SAM activity from the first to the second trial [*t*_(22)_ = 2.73, *p* = 0.037, Cohen's d = 1.046], but no difference involving the third trial. Post-hoc tests of the interaction effect, at a fixed level of presentation order, revealed that only during the first trial (trial 1), SAM activity decreased significantly from the first to the third segment [*t*_(132)_ = 3.635, *p* = 0.001, Cohen's d = 1.084], and also from the second to the third segment [*t*_(132)_ = 2.34, *p* = 0.042, Cohen's d = 0.697]. This is also shown in Supplementary Figure 6, and it is notable that, while activity significantly decreases only during trial 1, the overall activity is considerably higher than during the other trials. On the other hand, when fixing the time (segment), we found that SAM activity significantly decreased from the first trial to the second and third trial during the first segment (all *p*-values < 0.032). During the second segment, we only observed a significant decrease from the first to second trial (*p* = 0.01), also see Supplementary Figure 7. Combining these results, we can infer that SAM activity starts at a high level at the beginning of trial 1, and then rapidly drops until the end of the trial. During the following trials (2 and 3), SAM activity remains at a low level.

**Figure 9:**
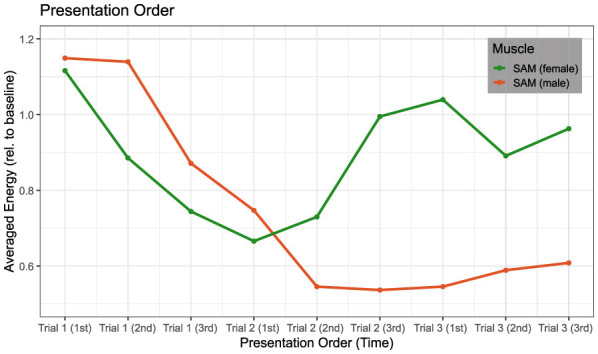
Averaged values of the superior auricular muscle (SAM), split according to the gender of the participants, re-arranged according to the presentation order (Trial 1, Trial 2, and Trial 3) and furthermore split into three parts of 90 seconds each, denoted by (1st), (2nd), and (3rd).

Interpretation of the significant three-way interaction between presentation order, time, and gender is more difficult due to the number of combinations. First, we compared the effect of gender at fixed levels of presentation order and time. We found that SAM activity in the female participants was significantly larger than in the male participants during trial 2 segment 3 and trial 3 segment 1, which appears somewhat spurious without the following context. Second, we compared trials at fixed levels of time and gender. We found that SAM activity was higher during trial 1, compared to trials 2 and 3 in almost all segments (only n.s. was trial 1/2 during segment 2, all other *p*-values < 0.05), but only in male participants. This implies that the decrease of SAM activity from the first to the second and third trial is driven by the male participants. Third, we compared segments at fixed levels of trial and gender. In male participants, we observed that during the first trial, SAM activity drops significantly from the first and second to third segment. In the second trial, a significant reduction is observed from the first to the second/third segment, i.e., a general within-trial reduction of SAM activity in the first and second trial (all *p*-values < 0.05). In female participants, we observed a significant reduction of SAM activity in the first trial (from the first to third segment), very similar to the male participants. However, in the second trial, SAM activity actually increased from the first to the third trial (*p*-values < 0.05), i.e., the opposite behavior of the male participants. Figure 9 displays this divergent behavior: in both, female and male participants, SAM activity initially decreases significantly during the first trial. However, in male participants, SAM activity decreases further and then remains at a low level, hence the significant difference between trials. SAM activity of female participants, on the other hand, appears to recover roughly to the baseline level (which also explains the two aforementioned instances of significant differences between male and female participants).

While the activity of the PAM might visually appear to increase during the third trial (Figure 8B), we found no significant effect of presentation order, time, gender, or an interaction effect between them.

At the corrugator, however, we can observe a strong increase with presentation order, see Figure 8C. In fact, presentation order and time, as well as their interaction were significant (all *p*-values < 0.01). Additionally, we observed a main effect of gender [*F*_(1, 22)_ = 5.325, *p* = 0.031], with corrugator activity in male participants being significantly larger (Cohen's d = 0.7). The main effect of presentation order suggests a significant increase from trial 1 to trials 2 and 3 (both *p*-values < 0.01). The main effect of time shows a significant increase between each segment (all *p*-values < 0.04), which is mostly identical to the data in Supplementary Figure 2, demonstrating a within-trial increase of corrugator activity. The interaction effect, for fixed levels of presentation order, revealed that in the first trial, each segment significantly increases (all *p*-values < 0.001), i.e., a monotonic increase of activity. In the second trial, differences between segments were not significant, and in the third trial, only the third segment is significantly larger than the first segment (*p* = 0.048), which is also displayed in Supplementary Figure 8. When fixing the level of time, we observed significantly less activity in trial 1 compared to trials 2 and 3, but only in the first two segments (all *p*-values < 0.003), see Supplementary Figure 9. Overall, we found that corrugator activity strongly increases during the first trial, and then remains at a constant, high level during the remaining trials.

**Figure 10:**
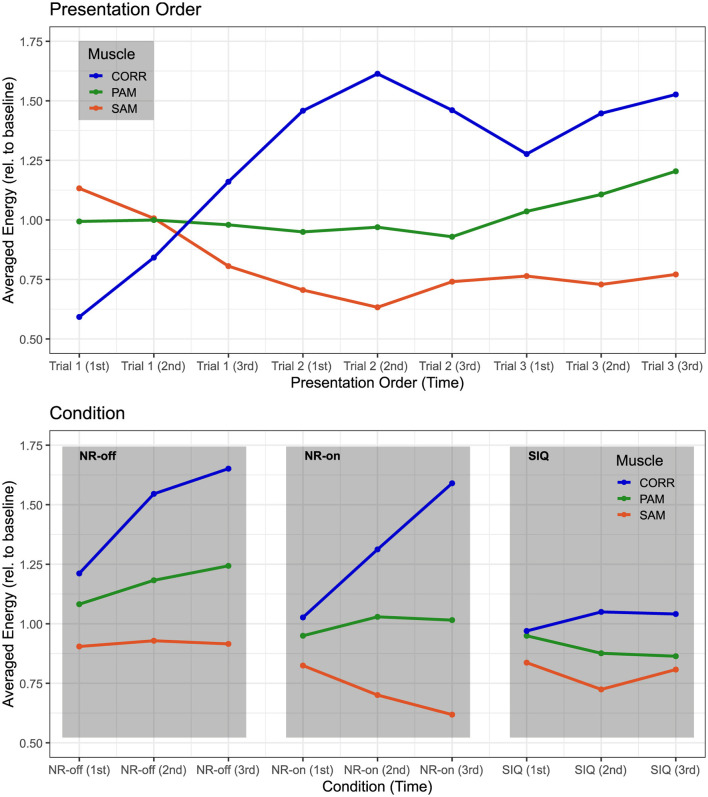
Averaged values of the posterior auricular muscle (PAM), superior auricular muscle (SAM), and corrugator supercilii (CORR). **Top**: Re-arranged according to the presentation order (Trial 1, Trial 2, and Trial 3). **Bottom**: Re-arranged according to the task difficulty condition (NR-off, NR-on, and SIQ). Note that each trial/condition is furthermore split into three parts of 90 seconds each, denoted by (1st), (2nd), and (3rd).

Figure 10 shows only the group averages, transformed back to the original scale, arranged in the presentation order (Figure 10 top) and according to the condition (Figure 10 bottom). When arranged in the presentation order, we can observe that the corrugator starts below the baseline level and then almost linearly increases until the middle of trial 2, at which point the activity remains relatively stable, highlighting the significant interaction effect. The PAM, as mentioned earlier, remains stable, perhaps slightly increasing during trial 3, but nevertheless, no statistical significance was observed. The SAM, on the other hand, starts approximately 12.5% above baseline level, but then drops and remains markedly below baseline for the remainder of the experiment. This not only explains the interaction effect, but also why averages during NR-off, NR-on, and SIQ in Figure 7A were on average below baseline. When arranged according to the condition (bottom plot), we see that the corrugator muscle strictly increases during NR-off and NR-on, but not during SIQ. At this point, it should be reiterated that the presentation order of the conditions were counterbalanced, and none of the conditions were over- or underrepresented. The PAM might slightly increase during NR-off and NR-on, and decrease during SIQ, but this effect was also not found to be significant. The SAM appears to be fairly stable during NR-off and SIQ, and might decrease during NR-on, but again, no significant interaction was observed. However, it is easy to see that the presentation order-related decrease of SAM activity is the cause for the below baseline activity during NR-off, NR-on, and SIQ. Additionally, when looking at the curves of the corrugator and SAM, when arranged in the presentation order, they appear to develop in opposite directions.

**Figure 11:**
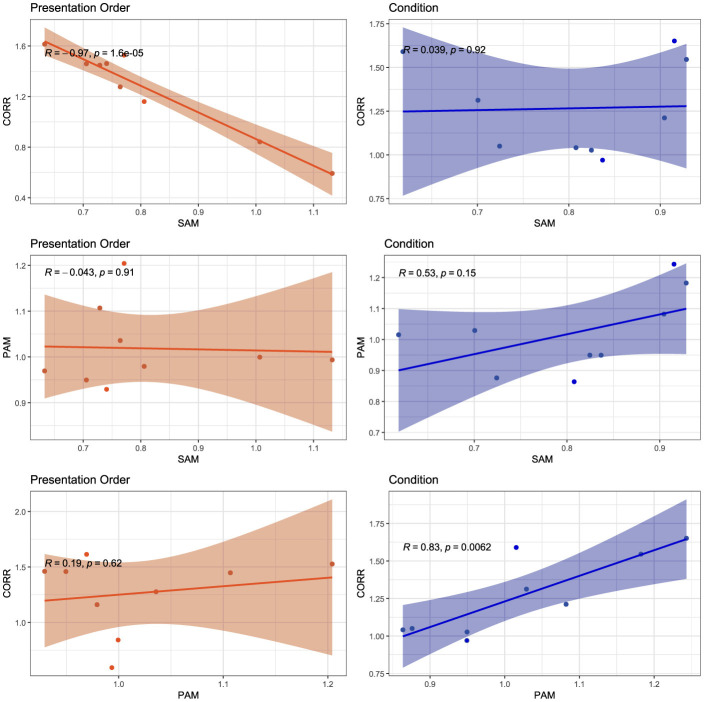
Correlation analysis between the posterior auricular muscle (PAM), superior auricular muscle (SAM), and corrugator supercilii (CORR). Data points are the same as in Figure 10.

This prompted us to calculate correlation coefficients, which are presented in Figure 11. When arranged in presentation order, corrugator and SAM are highly negatively correlated (*R* = −0.96, *p* < 0.001). When data were averaged according the condition, the correlation disappears (*R* = 0.039, *p* = 0.92). SAM and PAM, on the other hand, are not correlated to each other in either cases. While the PAM and corrugator are not correlated when in presentation order (*R* = 0.19, *p* = 0.62), they are positively correlated when arranged according to the condition (*R* = 0.83, *p* = 0.006), which is most likely because their activity increased during NR-off and NR-on (see Figure 10 bottom plot), even though this effect was not significant at the PAM.

## 4 Discussion

Activity of the SAM has previously been demonstrated to be significantly increased in normal-hearing participants when a large amount of listening effort was required to continuously attend a target speaker in noise (Schroeer et al., 2025a). At the same time, Schroeer et al. (2025a) did not observe a similar effect at the PAM, but instead found only a significant effect of the direction of the auditory stimuli. The significant dependence of auricular muscles activity on the direction of auditory stimuli has previously been explored (Strauss et al., 2020; Schroeer et al., 2023, 2025b), and was the reason why auditory stimuli were not spatially distributed in Schroeer et al. (2025a), as to avoid them as a confounding factor. The co-localization of all auditory streams (both target and distractor) is, however, a scenario which may not relate to real-life scenarios in a very meaningful way, which is why the current study utilized spatially distributed distracting noise. Additionally, as opposed to the relatively young (on average 28 years old), normal-hearing participants in Schroeer et al. (2025a), the current study recruited participants who were (mostly) considerably older as well as hearing aid users with at least one year of hearing aid experience, and measurements were conducted in a room at a hearing aid acoustician store instead of the lab setting described in Schroeer et al. (2025a). Furthermore, the manipulation of the required listening effort was not only performed by the inclusion of noise, but also by activating the hearing aid's noise reduction algorithm, which was specifically designed by the developer to reduce noise and to enhance speech clarity.

### 4.1 Subjective measures

As expected, self-reported listening effort increased significantly from SIQ to NR-off. Likewise, the use of the noise reduction algorithm (NR-on) was rated as intermediate, and significantly different from SIQ and NR-off, indicating a substantial reduction in perceived listening effort in a noisy environment. While inconsistencies across studies in tasks and methods make it difficult to directly compare studies related to hearing aids (Ohlenforst et al., 2017), these results concur with studies indicating that the use of a noise reduction algorithm can significantly enhance speech intelligibility and reduce listening effort (Bernarding et al., 2017; Picou and Ricketts, 2018; Fiedler et al., 2021; Valderrama et al., 2025). Distractibility ratings (how often participants lost the target stream), paint a similar picture: activating the noise reduction algorithm enabled participants to follow the target stream with a significantly higher consistency in a noisy environment. While the separation between conditions is not quite as extreme compared to the perceived listening effort, we believe that this metric provides an easy way to interpret the benefit of the noise reduction algorithm. Winn and Teece (2022) found that mentally repairing and reconstructing misheard words significantly increased listening effort. This could explain the similarity between the self-reported listening effort and distractibility: as participants lost the target stream most frequently during NR-off, “finding” the target stream again and reconstructing missing segments (e.g., through contextual information) could have been perceived as very effortful.

### 4.2 Electromyographic measures

Electromyographic data obtained from the auricular muscles only partially met our expectations. While the PAM was not affected by the increased task demand in Schroeer et al. (2025a), in the current study, we observed a significant increase in PAM activity from SIQ to NR-off, i.e., from the least to the most effortful condition. While PAM activity during NR-on was not significantly different from NR-off or SIQ, their values appear to be at an intermediate level, generally reflecting the subjective ratings. Activity of the SAM, however, was not significantly affected by the three conditions. While there may be a non-significant trend of a lower SAM activity during NR-on compared to NR-off (Figure 7A), activity of the SAM measured in the current group of hearing aid users does not support our research hypothesis, despite the apparent subjective effect.

It is worth noting some important differences between the current study and Schroeer et al. (2025a). In Schroeer et al. (2025a), the difficulty of the conditions were manipulated by the amount and intensity of the distractor streams, with the SNR changing from +10 dB, to +2 dB, and finally to -2 dB in the most effortful condition. In the current study, the least effortful condition was SIQ, which would correspond to an SNR of +∞, compared to +10 dB in Schroeer et al. (2025a). On the other end of the spectrum, in the most effortful condition of the current study (NR-off), noise was presented at -2 dB SNR, which is the same as the SNR in the “difficult” condition in Schroeer et al. (2025a), albeit with a spatial separation between target and distractor streams. Comparison of the “intermediate” conditions is, however, not as straightforward. Schroeer et al. (2025a) adjusted the amount and intensity of the distractor streams to achieve a +2 dB SNR. In the current study, the intensity of the audio files during NR-on were identical to the NR-off condition (–2 dB SNR). A reduction in listening effort was aimed for by the noise reduction algorithm (see 2.3) instead of a direct adjustment of the acoustic soundscape. This algorithm manipulates the signal in two important ways. Mainly, it identifies and removes noise, increasing the SNR by up to +10 dB. In such an ideal scenario, the SNR during NR-on would then be at +8 dB, fairly close to the “easy” condition in Schroeer et al. (2025a). In addition to the SNR changes, the remaining signal receives a boost to compensate the energy loss due to the noise reduction, which would result in a higher intensity of the (target) podcast during NR-on, compared to NR-off and SIQ.

Overall, the NR-off and the “difficult” condition in Schroeer et al. (2025a) are fairly comparable from an acoustical point-of-view. However, in noisy environments, older, hearing impaired adults can also perceive more listening effort compared to younger, normal-hearing adults (Anderson Gosselin and Gagné, 2011; Alexander, 2021), even though spatially separating speech from noise decreases the difficulty (Arbogast et al., 2005). Therefore, the NR-off condition, considering the participant group, could have been considerably more effortful, which could also be concluded by the observation that the self-reported LE scores are at the upper end of the 7-point scale. SIQ could be considered less effortful than the “easy” condition from Schroeer et al. (2025a). However, while subjective ratings were very low during SIQ, the distractibility rating was far from being scored on the lowest end of the scale. This indicates that even during SIQ, participants struggled with maintaining their focus on the target, even though no competing sound sources were present. Even normal-hearing participants can experience a substantial amount of listening effort in a quiet environment when speech is not sufficiently loud (Husstedt et al., 2025), and for hearing aid users, perceived listening effort in quiet can be even larger compared to normal-hearing participants (Ferschneider and Moulin, 2023) (even though hearing aids aim to compensate for this difference), which means that SIQ should not necessarily be considered completely effortless.

This could indicate that the current study stretches over a larger difficulty range (the difference between least and most effortful condition) than Schroeer et al. (2025a), based on the acoustics and the participant group. While the subjective measures clearly locate the NR-on condition at an intermediate level, potentially closer the NR-off than SIQ based on the subjective LE scores, activity of the PAM behaves similar, but with an important distinction: While there is a significant difference between PAM activity during NR-off and SIQ, the intermediate NR-on condition is not significantly different from either. So while PAM activity appears to be at an intermediate level, there is not enough contrast to clearly demonstrate an effect of the noise reduction algorithm (from the perspective of the NR-off condition), but also not enough to find a difference between no noise and a moderate noise level (from the perspective of the SIQ condition). However, a similar lack of contrast between (subjectively) clearly different conditions based on EMG data have also been reported by Mackersie and Cones (2011) and Schroeer et al. (2025a), using the frontalis muscle and SAM. Nevertheless, while the current data points toward the PAM as being affected by different levels of effortful listening, a similar behavior was not observed in Schroeer et al. (2025a). A possible explanation could be related to the fact that in the current study, the acoustic soundscape contained spatially distributed noise. Auricular muscles in humans are very much influenced by the spatial location of auditory stimuli in a complex pattern, with a preference for stimuli outside the participant's field-of-view (Strauss et al., 2020; Schroeer et al., 2024, 2025b,a). As the PAM has consistently displayed an increased activity in the direction of lateralized sounds (during both, endogenous and exogenous modes of attention), we could hypothesize that the PAM is primarily a “spatial indicator” of the auriculomotor system. In this case, in the absence of spatially distributed or lateralized sounds, the auriculomotor system might not engage the PAM (or only to a lesser degree). This would explain the unexpected inactivity of the PAM in Schroeer et al. (2025a), as well as the low activity during SIQ in the current study. During NR-off, due to the high spatial complexity of the acoustic scene, PAM activity would be significantly increased. The noise reduction algorithm used during NR-on, as it reduces the spatially distributed noise, would then only lead to a small increase of PAM activity (relative to SIQ).

However, it should also be noted that in order to understand speech in noise, effects such as binaural unmasking and grouping (in particular segregating speech from noise) are extremely important (Bronkhorst, 2015). During SIQ, the underlying neurophysiological processes are likely not (or less) engaged, complicating a direct comparison between SIQ and the other conditions.

The most unexpected difference between the current results and Schroeer et al. (2025a) was observed at the SAM. While Schroeer et al. (2025a) found a significant increase of SAM activity in the most effortful condition, we were unable to find a similar effect (potentially a trend, but no significance was reached). However, a large confounding issue we encountered in the current study was the significant decrease of SAM activity during the first trial. This decreased activity persisted throughout the experiment, at least in the male participants, while SAM activity of female participants appeared to recover (Figure 9, which should, due to the small sample size, be interpreted with caution). Even tough the randomized presentation order of the experimental conditions was implemented to mitigate such presentation order-related effects, the decrease is most likely responsible for the observation that the averaged SAM activity in all three conditions was below baseline level, which may mask effort-related effects and will be discussed separately in 4.3.

The last muscle which was recorded, the corrugator supercilii, displayed a significant time-dependent increase of activity, but only during NR-off and NR-on, whereas corrugator activity remained at a significantly lower level during SIQ. As there was no significant difference between NR-off and NR-on, a ceiling effect might have been reached, similar to the results reported by Lau et al. (2019) using pupil dilation.

Mackersie and Cones (2011), as mentioned before, observed a significant increase at the frontalis muscle in an increasingly demanding listening task. The frontalis muscle, a muscle which is close to, but also considerably larger than the corrugator, could be a source of cross-talk to the electrodes intended to record corrugator activity, or they might simply react similarly during an effortful task. For example, Cohen et al. (1992) found that activity of the frontalis and of the corrugator muscle increased significantly when a pitch discrimination task became more difficult. However, Francis et al. (2021) was unable to observe a significant effect of corrugator activity in challenging listening conditions, indicating that corrugator activity does not necessarily has to correlate with an increasing task demand.

We should, however, also consider the possibility that the increased activity of the corrugator, which is likely due to furrowing of the eyebrows, could be a generalized, non-specific reaction to the increased task demand, and not related specifically to an auditory task. For example, Waterink and Van Boxtel (1994) observed a gradual increase in EMG activity obtained from the frontalis and corrugator muscles during a visual task, indicating exerted mental effort. Additionally, Van Boxtel and Jessurun (1993) observed that frontalis and corrugator activity increased significantly with time (time on task), but this effect was particularly strong when the task became more difficult over time. Importantly, the authors found no significant difference between a visual and an auditory task, indicating that these muscles are independent of a particular modality. On the other hand, the current task was purely an auditory task, as participants were instructed to look at a fixation cross, i.e., the visual modality remained a constant and low effort task. Nevertheless, these studies furthermore demonstrate, as mentioned before, that the frontalis and corrugator muscles can display the same behavior during an effortful task. Ultimately, the increased activity of the corrugator muscle could be attributed to the perceived listening effort, but previous studies indicated that this might be more of a generalized response to a difficult task, regardless of stimulus modality. Therefore, to be potentially utilized as a proxy of listening effort, all other modalities would have to be tightly controlled.

Nevertheless, the intended purpose behind recording the corrugator muscle in the current study was mainly to ensure that signals obtained from auricular muscles were not the result of interference from facial muscles, which are expected to be more active in a demanding task, as mentioned before. This concern was raised in particular as a potential confounding factor to the recorded SAM activity in Schroeer et al. (2025a), as studies have reported mixed results concerning the co-activation between facial and auricular muscles (Bérzin and Fortinguerra, 1993; Strauss et al., 2020; Rüschenschmidt et al., 2022; Lipede et al., 2023).

Lastly, it is important to point out that the PAM is the only muscle which was recorded during the experiment, which did not display a significant effect when re-arranged according to the presentation order. Therefore, the current data support that the activity of the PAM in hearing aid users is affected by listening effort, as (a) we observed a significant difference between the most and the least effortful condition, and (b) we observed no effect of the presentation order. Both of these properties are shared by the subjectively perceived listening effort of the participants.

### 4.3 Potential confounds

The largest confounding factor is the very strong, time-dependent drift in EMG activity, most importantly a strong decrease of SAM activity during the first trial (Figure 10).

This drift at the SAM is markedly different from results of previous studies. In the endogenous attention experiment of Strauss et al. (2020), SAM activity remained fairly stable over the course of their experiment (as opposed to the PAM, which only slightly decreased over time). In Schroeer et al. (2025a), SAM activity also remained mostly stable during the easy and medium conditions, but increased with time in the most difficult condition. Additionally, Schroeer et al. (2025a) found no presentation order-related decrease of SAM activity. While we counterbalanced the presentation order, and no condition was over- or underrepresented, it is likely that this effect was “bleeding” into all three conditions, at least the more sustained part, which was predominantly observed in male participants. The initial rapid decrease (which we observed in both genders), is not visible in all conditions (see Figure 10), and might have been successfully “averaged out.” This is not only the explanation as to why SAM activity is mostly below baseline level (Figure 7), but might also mask effort-related effects. This can be strengthened by the fact that Schroeer et al. (2025a) did not find a similar presentation order effect.

As mentioned earlier, a major difference between the current experiment and Schroeer et al. (2025a) was the presence of spatially distributed sound sources, which have a strong effect on the activity of auricular muscles, in particular the PAM (Strauss et al., 2020; Schroeer et al., 2025b). At the same time, Schroeer et al. (2024) found that, under certain circumstances, the PAM and SAM can display a complex, partially antagonistic relationship: while the PAM activity increases in response to transient auditory stimuli, SAM activity is instead suppressed. Even though it is currently not supported by our data, we could speculate that the decrease of SAM activity could potentially be caused by a similar mechanism. While the PAM is an unlikely candidate, as PAM activity does not increase with time, we found a significant negative correlation between the SAM and corrugator muscle when arranged according to the presentation order. Even though we cannot establish causation, we could speculate that there might be a similar suppression effect mediated by the corrugator muscle (or perhaps another facial muscles which we have not recorded) to the SAM. However, this would have to be carefully examined in a more controlled, dedicated experiment (including the apparent gender-related difference regarding the recovery of SAM activity which we observed).

Another notable difference between the current study and Strauss et al. (2020) as well as Schroeer et al. (2025a) is the way in which participants were positioned and seated. In Strauss et al. (2020) and Schroeer et al. (2025a), participants placed their head on a chin-rest and were therefore slightly leaning forward. In the current study, participants were sitting upright with their heads gently fixated at the temples to stabilize their head position (see Figure 3). It is possible that holding his position could be overall more strenuous, and in an attempt to sit as upright and straight as possible, participants could have exerted more muscular tension in the beginning of the experiment, and started to gradually relax (or become more tired) over time, resulting in a decrease of EMG activity. This possibility could be compounded by the fact that the participants of the current study were, on average, considerably older compared to the participants in Strauss et al. (2020) and Schroeer et al. (2025a), and were furthermore not able to take small breaks between conditions. While this effect might, in isolation, be attributed to fatigue, and maybe partially to the age of the participants, it does not explain why the PAM and corrugator muscles do not share a similar response. In fact, we found that activity of the SAM and corrugator are significantly negatively correlated (Figure 10). This is a perplexing finding because, if anything, we would have expected a positive correlation, either due to volume conduction or co-activation (the SAM may be more active or remain unchanged during a furrowing of the eyebrows, see Bérzin and Fortinguerra, 1993; Rüschenschmidt et al., 2022; Lipede et al., 2023), but to the best of our knowledge, we found no publication describing a negative correlation between these two. Furthermore, the correlation was no longer present when data were ordered according to their condition, reinforcing that the randomized presentation order was generally successful.

While we are not able to definitely determine the cause of the decreasing SAM activity, it is an unexpected and concerning observation that should be carefully considered when planning future experiments as it is likely to mask smaller, but more interesting effects.

Furthermore, by the apparent lack of positive correlation between SAM and corrugator (when data are arranged in presentation order), we are able to conclude that the signals obtained from electrodes at the SAM are probably not due to volume conduction from the corrugator muscles, which likely increased because participants started to furrow their eyebrows during the noisy, more effortful listening conditions. The PAM is located even further away from the corrugator muscles (and other facial muscles) than the SAM. In approximate terms, the SAM is located somewhere in the middle of an imaginary line connecting the corrugator and the PAM. It is therefore also unlikely that the increased PAM activity during NR-off (see Figure 7) is due to corrugator activity, as we would expect the SAM to be contaminated as well. Overall, while we observed an unexpected (and undesired) decrease of SAM activity, this coincidentally allows us to argue against cross-talk from the corrugator on the SAM, and by extension, on the PAM as well.

Lastly, we should consider the possibility that the presentation order related increase of corrugator activity might also have influenced the condition effect, even though the time course of the corrugator is very different during SIQ compared to NR-off and NR-on. In addition to the randomized presentation order, we can look at the results of Van Boxtel and Jessurun (1993). In their study, the authors found that during tasks with a constant difficulty (or mental effort), corrugator (and frontalis) activity increased significantly with time. However, when the task difficulty increased dynamically over time, this effect was even larger, indicating that the corrugator is sensitive to the time on task in addition to the task difficulty. The randomized presentation order may then have largely negated the time on task effect (Figure 10 top), allowing us to at least partially isolate the effect of effort (Figure 10 bottom).

## 5 Conclusion

The current study examined the effects of effortful listening on the activity of auricular muscles in hearing aid users in three different conditions, which differed in task demand. The demand was increased by the addition of realistic, spatially distributed noise, and an intermediate level was achieved by activating a noise reduction algorithm.

Perceived listening effort and distractibility (how often they lost the target stream) were significantly increased by the addition of noise, but could also be reduced by the activation of the noise reduction algorithm to a distinct intermediate level. Activity of the PAM generally followed this pattern: PAM activity was highest during NR-off and lowest during SIQ, but activity of the PAM was not sensitive enough to find a significant effect of the noise reduction algorithm. Activity of the corrugator muscles significantly increased during the NR-off and NR-on conditions, to the point to where they significantly different from SIQ, but not from each other, which could be related to the perceived listening effort (with a ceiling effect), but also be more of a general indicator to task-related effort. Activity of the SAM was not affected by the experimental condition, but we observed a strong and sustained decrease of EMG activity during the course of the experiment (mostly in male participants), which likely masked any potential effort-related effects.

Overall, while some of the results are encouraging for the potential future use of auricular muscles as an objective indicator of effortful listening in hearing aid users, important factors, such as the acoustic context of the experiments, baseline drifts, fatigue effects, and contamination from facial muscles need to be considered more carefully, as they may explain some of the unexpected outcomes compared to Schroeer et al. (2025a).

## Data Availability

The raw data supporting the conclusions of this article will be made available by the authors, without undue reservation.
