# Supplementary material for: Electromyographic responses of the auriculomotor system during effortful listening in hearing aid users
Source: Front Neurosci. 2026 Aug 4;20:1838748. doi: 10.3389/fnins.2026.1838748 (PMC13481888; doi:10.3389/fnins.2026.1838748)
Supplement: Supplementary file 1 [file Data_Sheet_1.pdf]

Supplemental Material For: Electromyographic Responses of the Auriculomotor System during Effortful Listening in Hearing Aid Users

Andreas Schroeer[1,2], **Tanja Biehl**[1,4], **Matthias Latzel**[3], **Stefan Launer**[3], **Daniel J. Strauss**[1,2], **and Farah I. Corona-Strauss**[1,2]

[1]**Systems Neuroscience and Neurotechnology Unit**, Faculty of Medicine, Saarland University & htw saar, Homburg/Saar, Germany

[2]**Center for Digital Neurotechnologies Saar**, Homburg/Saar, Germany

[3]**Sonova AG**, Stäfa, Switzerland

[4]**Auris Hörcenter GmbH**, St. Wendel, Germany

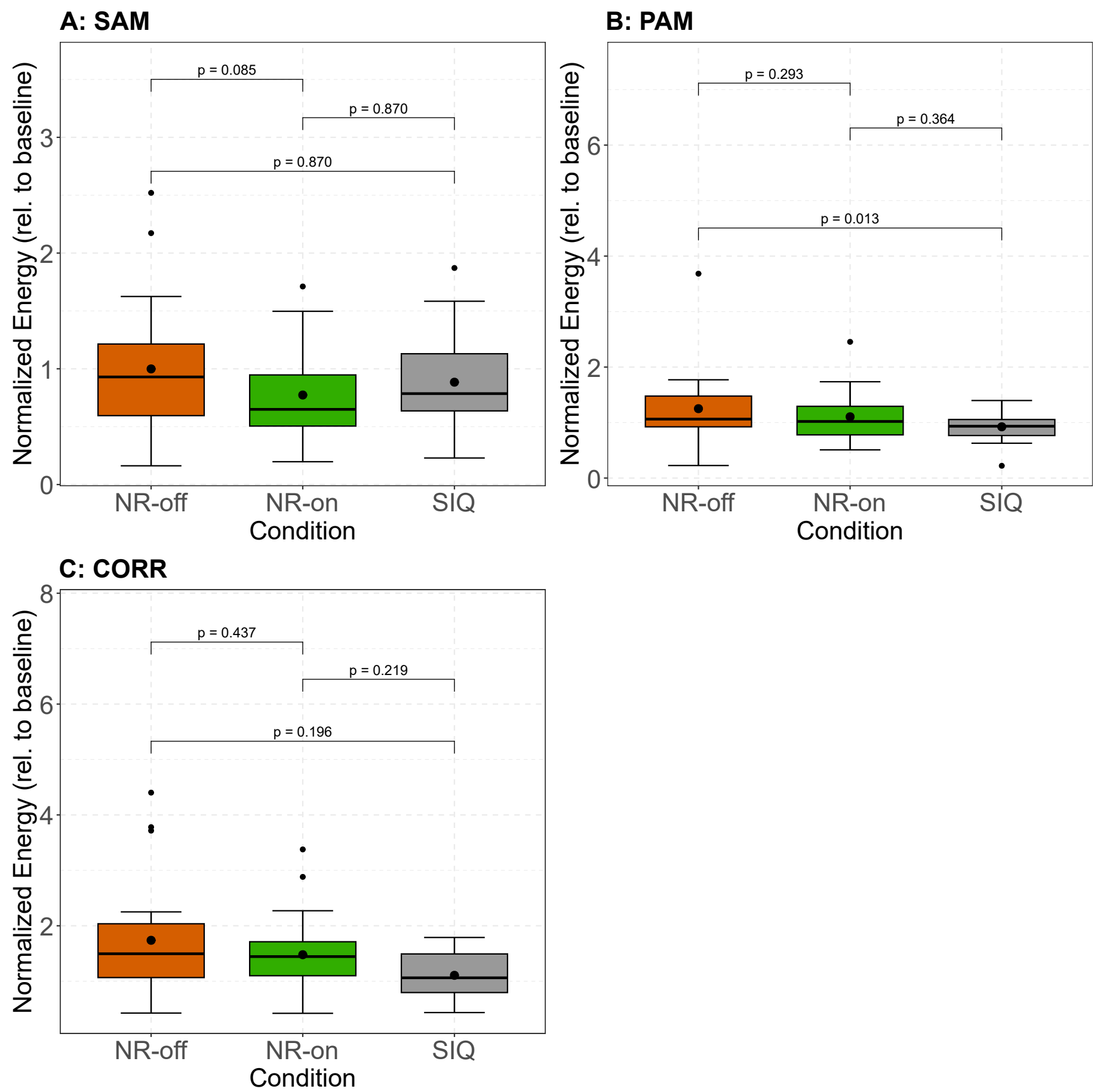

Supplemental Figure 1: Boxplots of the averaged, normalized activity of the SAM, PAM, and corrugator muscles in all three recorded conditions. The λ values in the y-axes indicate the parameter used in the Box Cox transform in eq. (1). P-values were calculated using Bonferroni-Holm corrected t-tests.

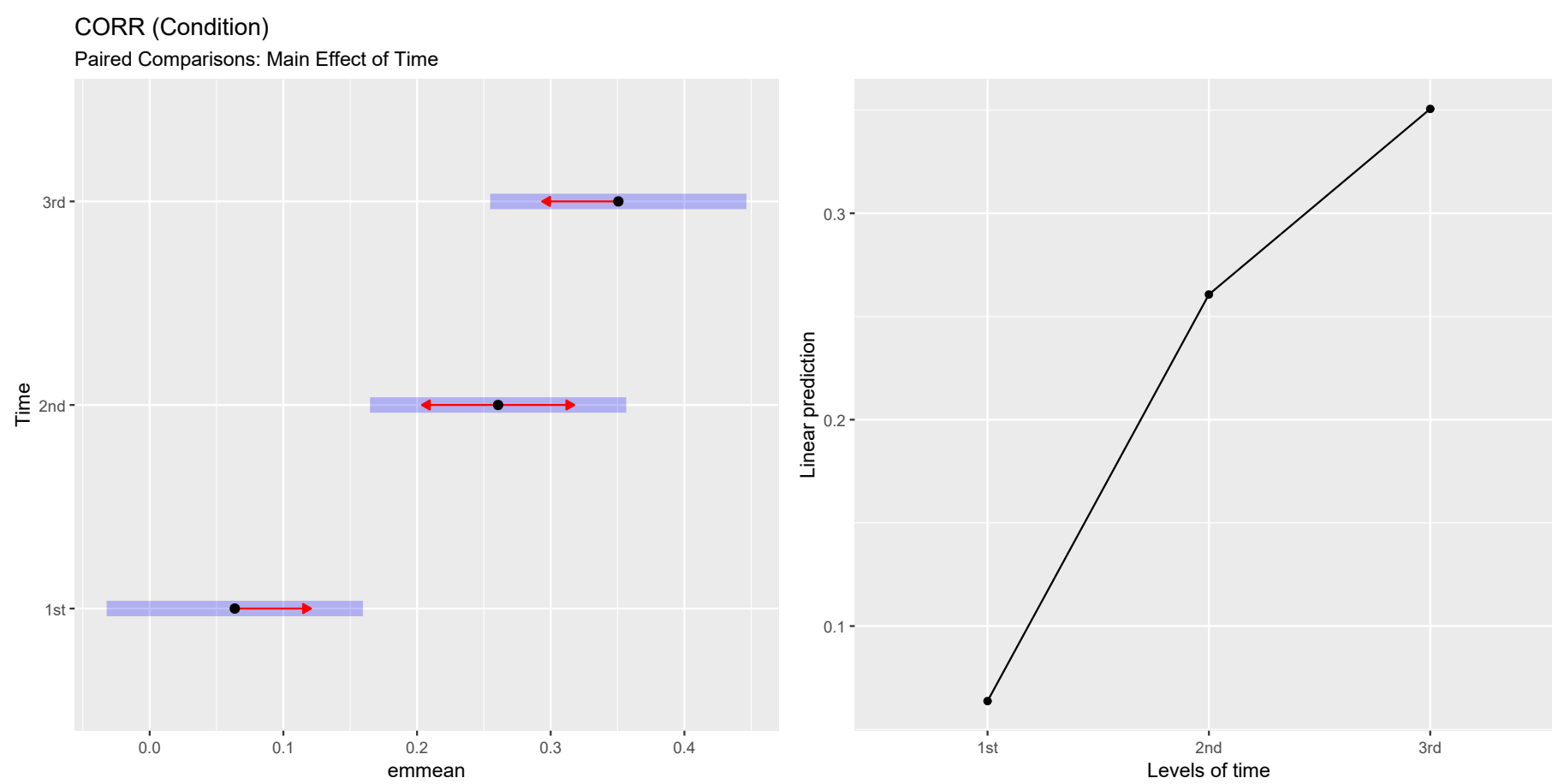

Supplemental Figure 2: Data displaying the significant main effect of time on the corrugator muscle (CORR). Black dots indicate mean values, blue areas mark 95% CI intervals. Red arrows are scaled so if they do not overlap, pairwise differences are significant.

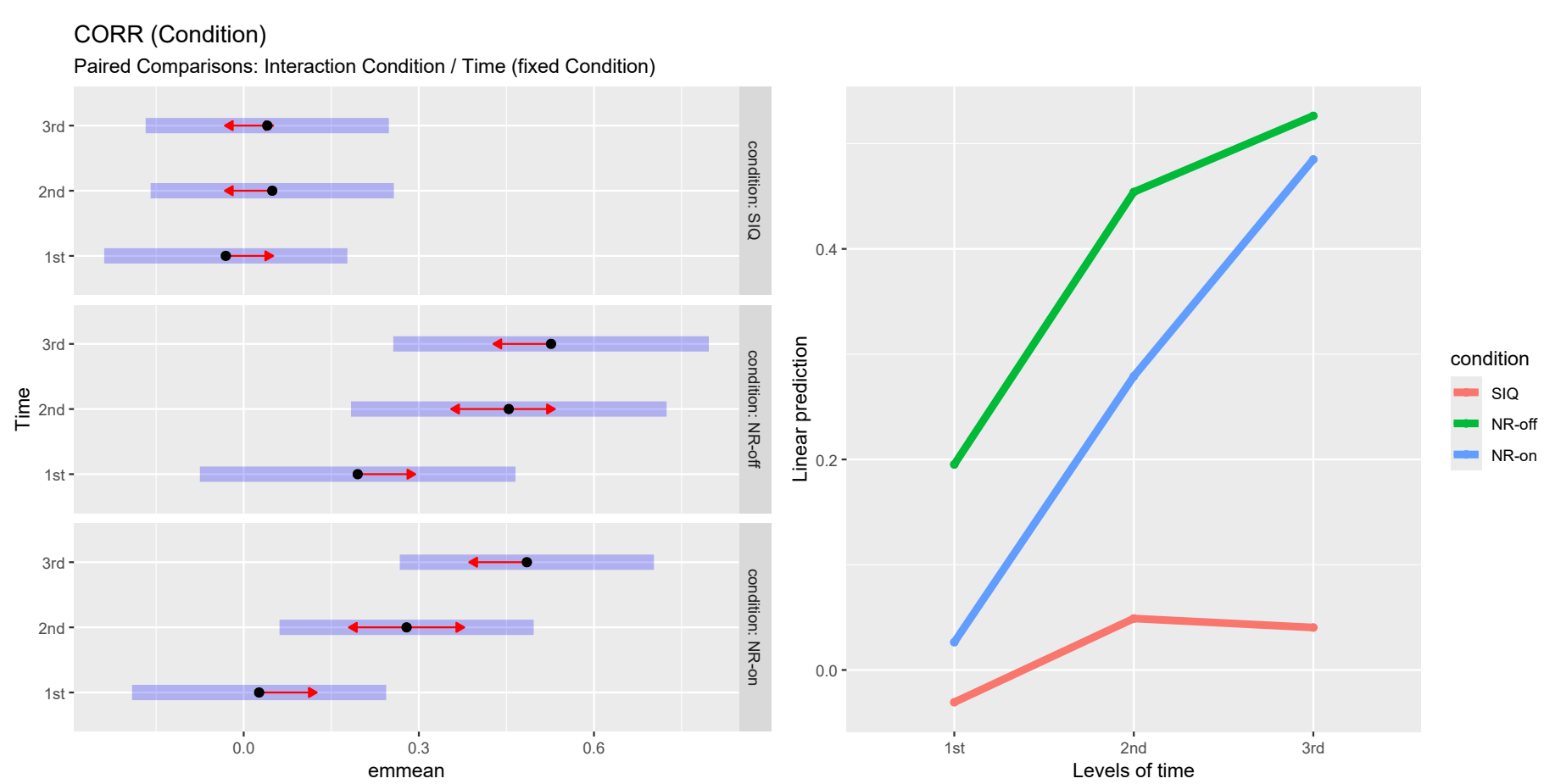

Supplemental Figure 3: Data displaying the significant interaction effect between time and condition, for fixed levels of condition on the corrugator muscle (CORR). Black dots indicate mean values, blue areas mark 95% CI intervals. Red arrows are scaled so if they do not overlap, pairwise differences are significant.

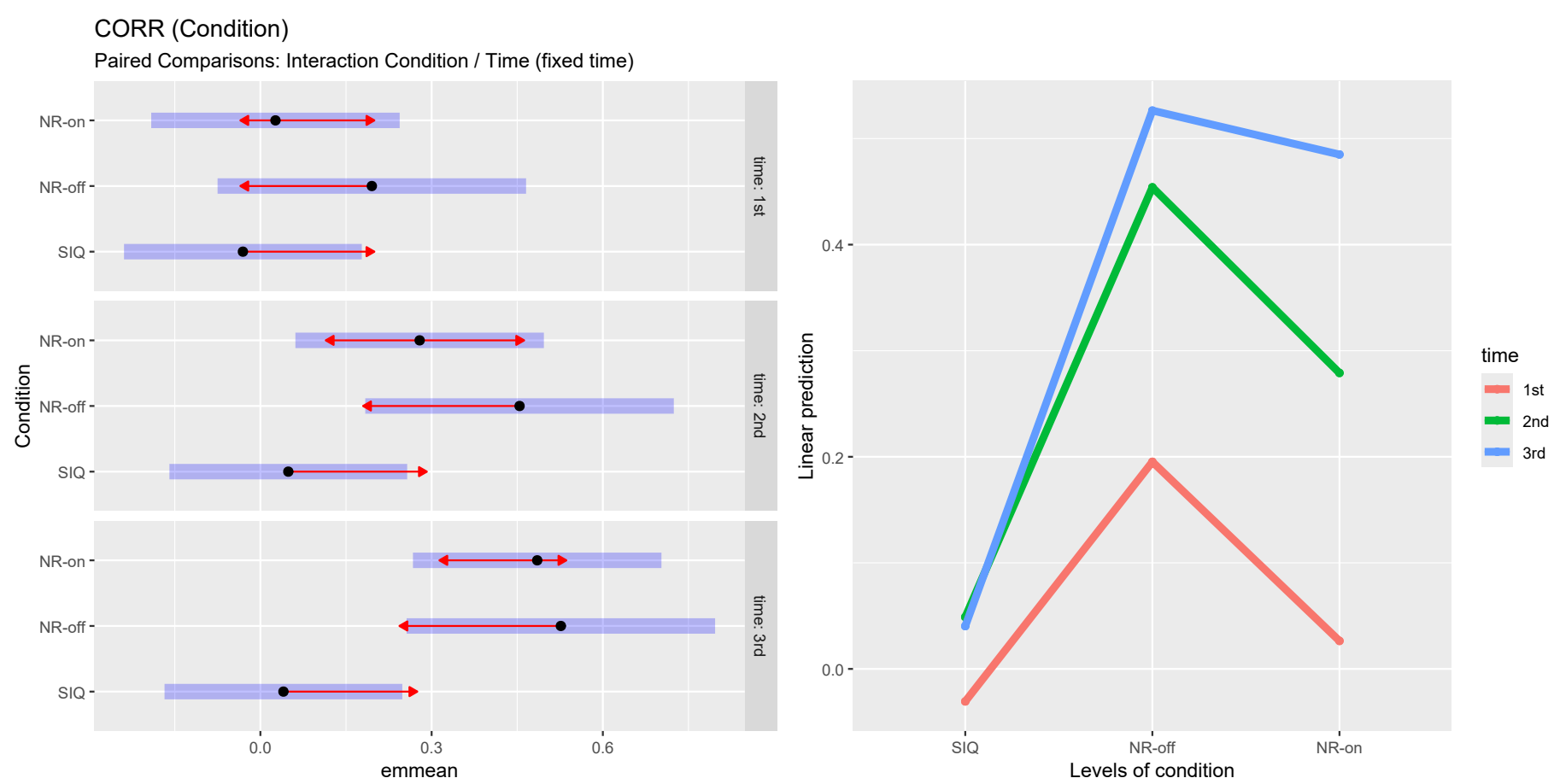

Supplemental Figure 4: Data displaying the significant interaction effect between time and condition, for fixed levels of time on the corrugator muscle (CORR). Black dots indicate mean values, blue areas mark 95% CI intervals. Red arrows are scaled so if they do not overlap, pairwise differences are significant.

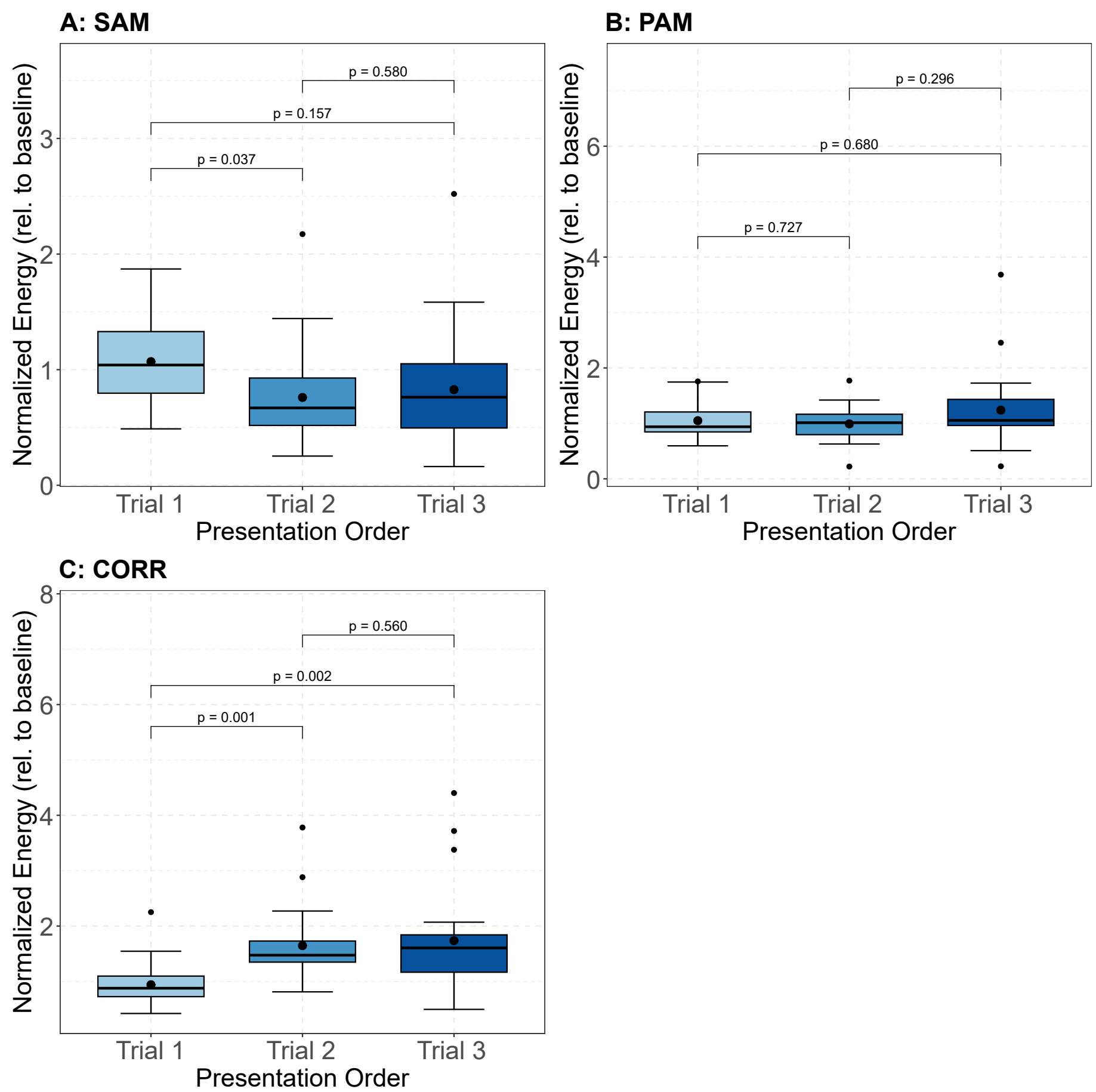

Supplemental Figure 5: Boxplots of the averaged, normalized activity of the SAM, PAM, and corrugator muscles when re-arranged according their presentation order. The λ values in the y-axes indicate the parameter used in the Box Cox transform in eq. (1). P-values were calculated using Bonferroni-Holm corrected t-tests.

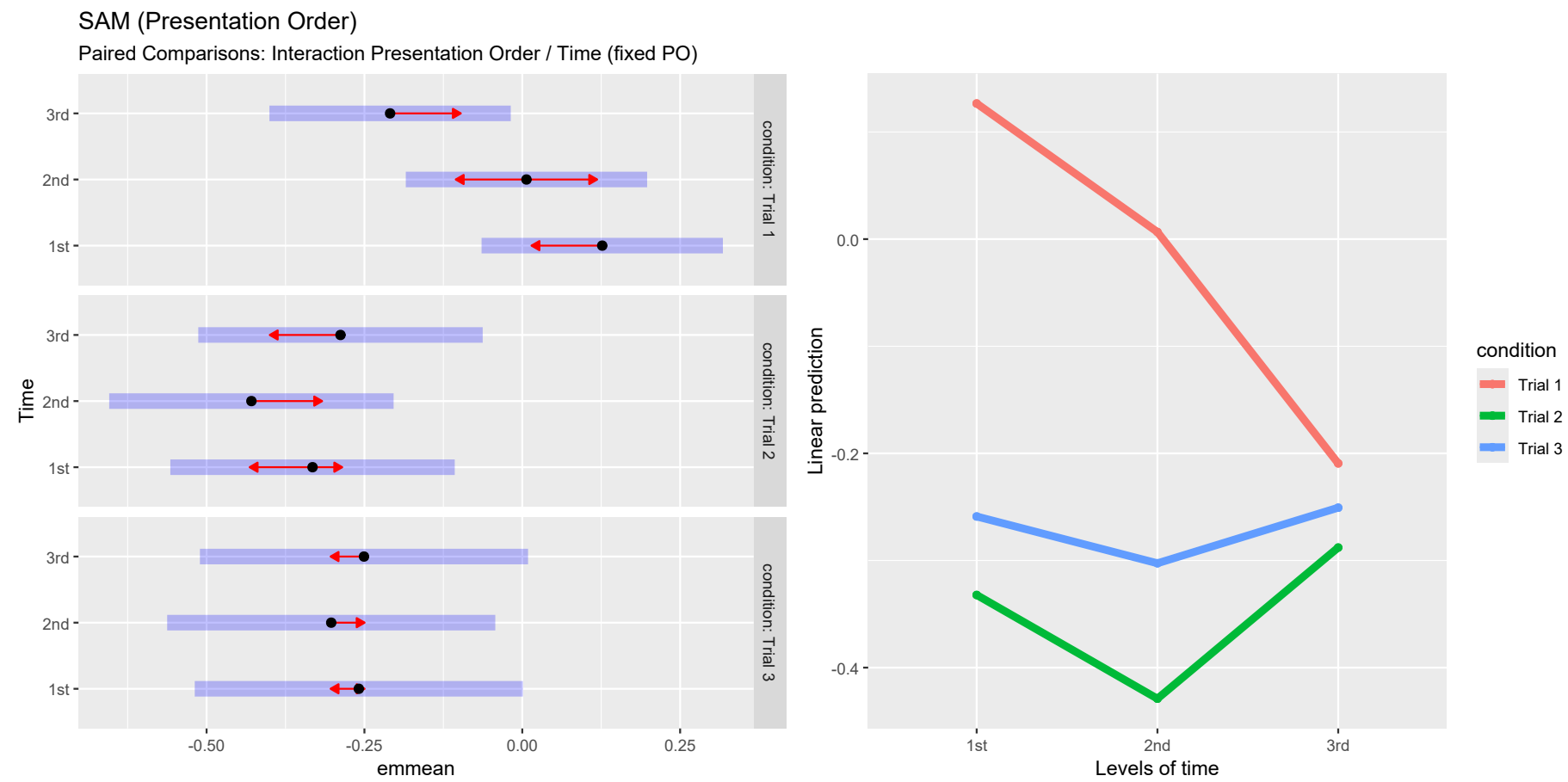

Supplemental Figure 6: Data displaying the significant interaction effect between time and presentation order, for fixed levels of presentation order on the superior auricular muscle (SAM). Black dots indicate mean values, blue areas mark 95% CI intervals. Red arrows are scaled so if they do not overlap, pairwise differences are significant.

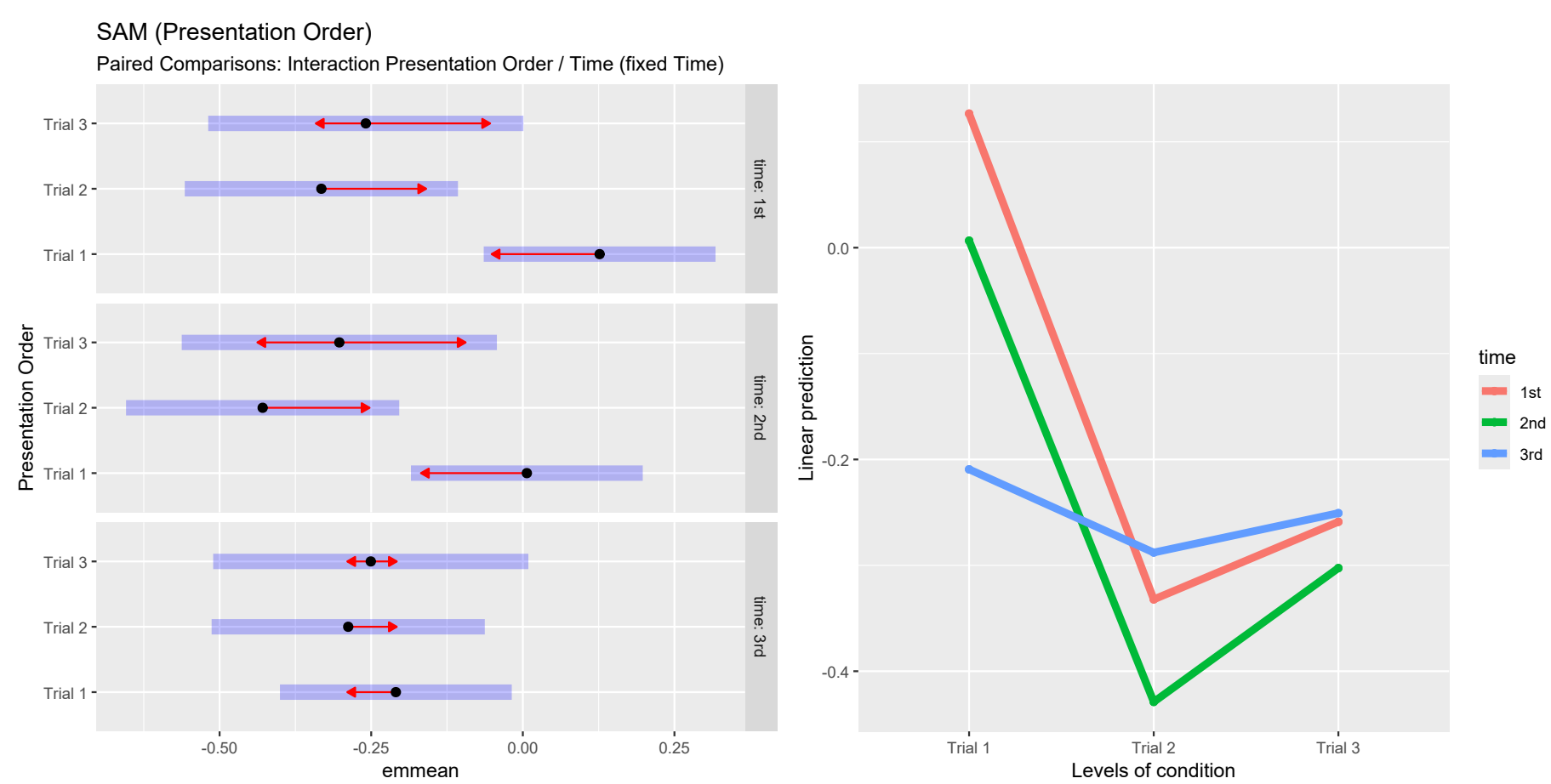

Supplemental Figure 7: Data displaying the significant interaction effect between time and presentation order, for fixed levels of time on the superior auricular muscle (SAM). Black dots indicate mean values, blue areas mark 95% CI intervals. Red arrows are scaled so if they do not overlap, pairwise differences are significant.

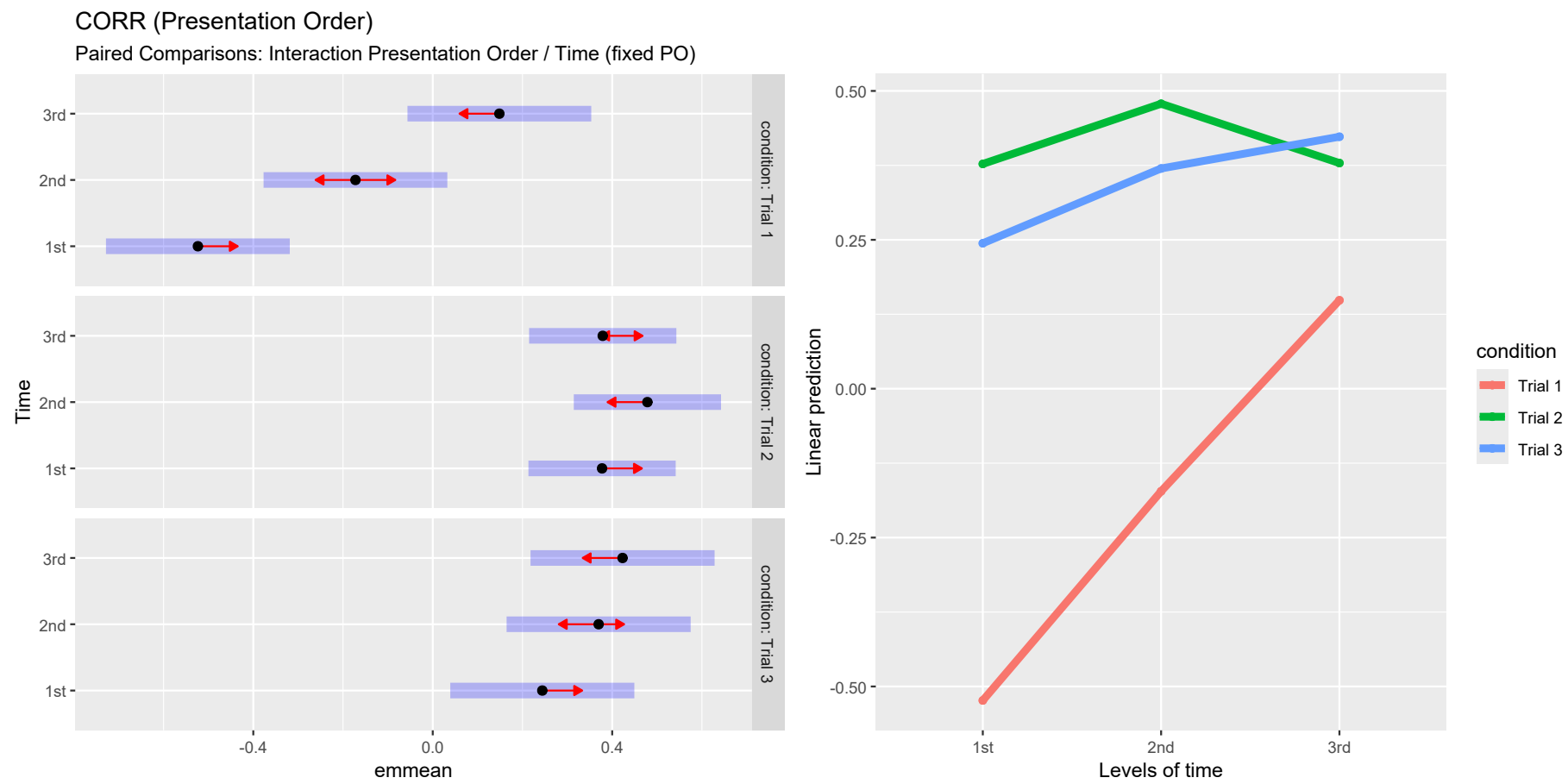

Supplemental Figure 8: Data displaying the significant interaction effect between time and presentation order, for fixed levels of presentation order on the corrugator muscle (CORR). Black dots indicate mean values, blue areas mark 95% CI intervals. Red arrows are scaled so if they do not overlap, pairwise differences are significant.

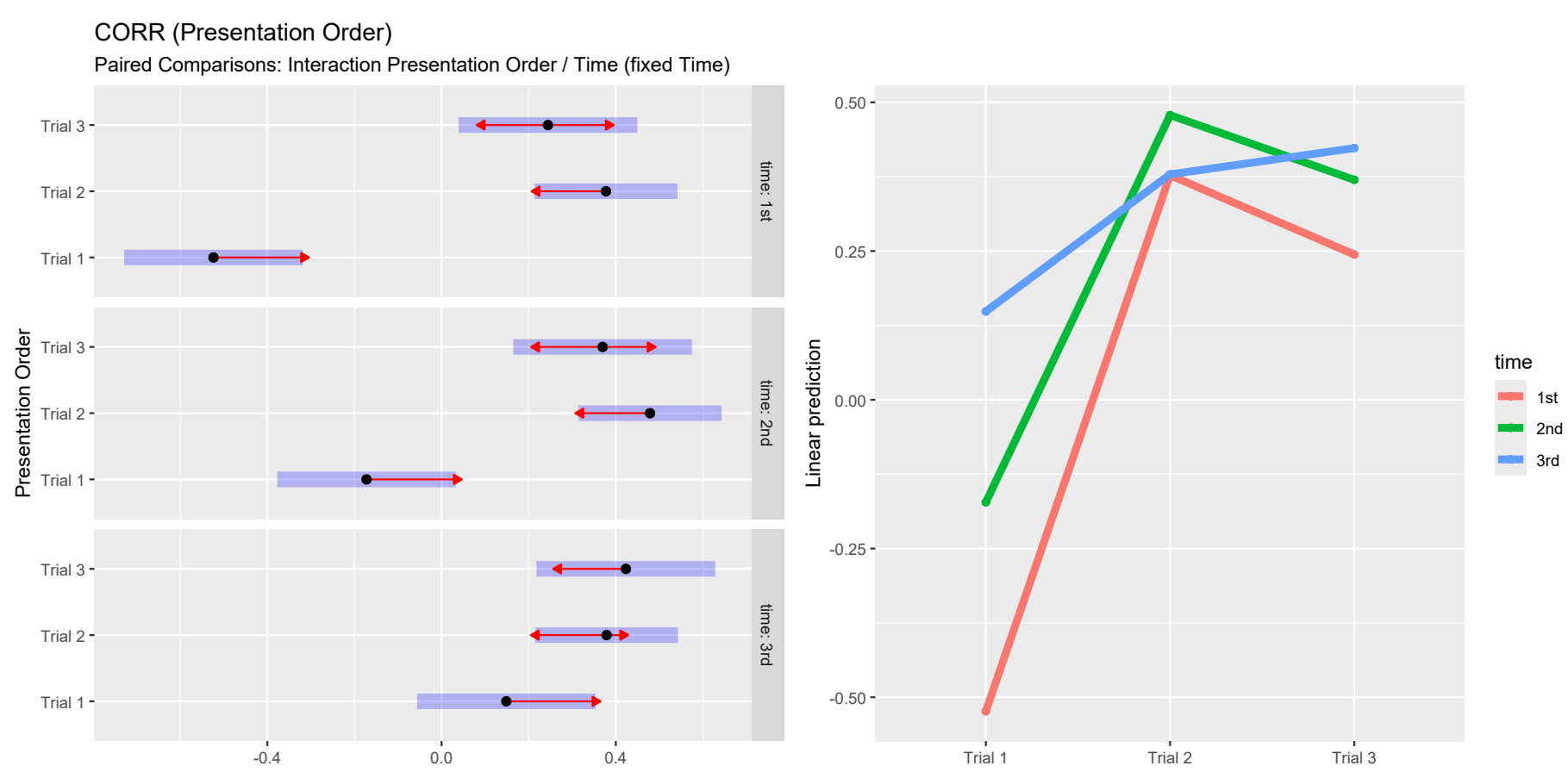

Supplemental Figure 9: Data displaying the significant interaction effect between time and presentation order, for fixed levels of time on the corrugator muscle (CORR). Black dots indicate mean values, blue areas mark 95% CI intervals. Red arrows are scaled so if they do not overlap, pairwise differences are significant.

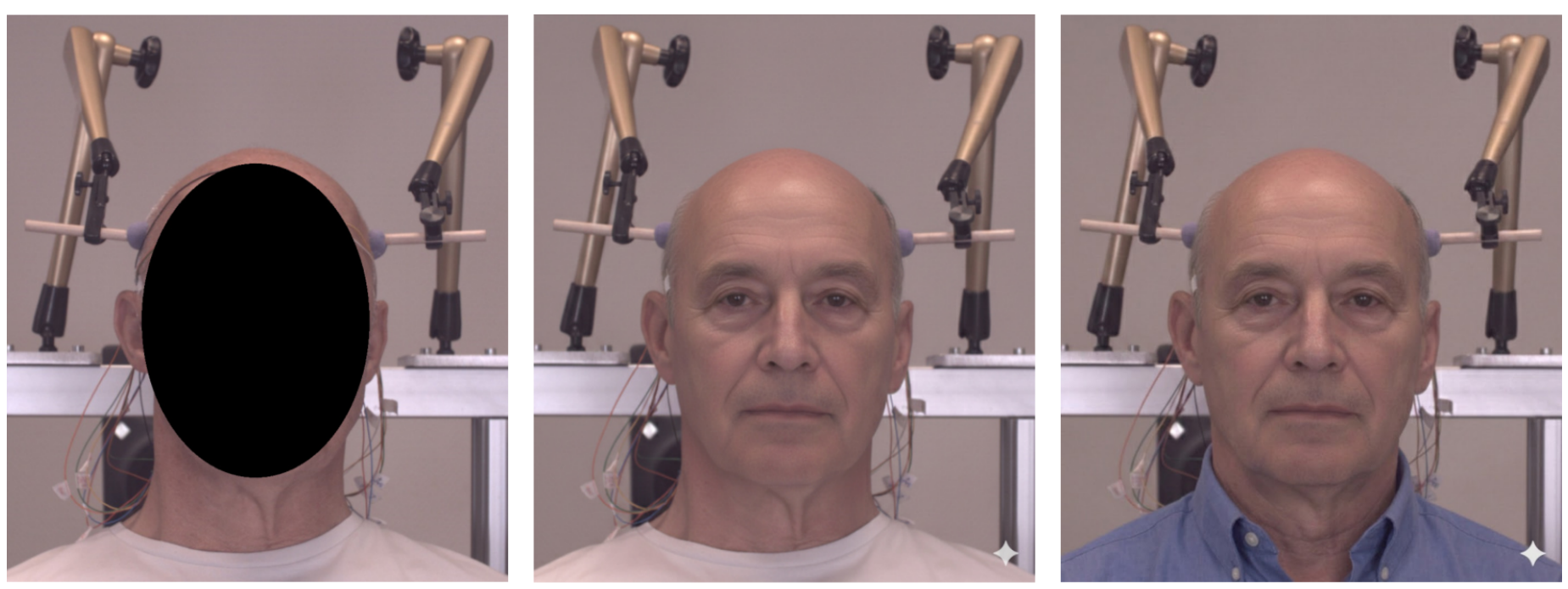

Prompt: Fill the blank spot with a face. | Prompt Replace the the shirt. | Final image.

Supplemental Figure 10: Prompts provided to Gemini 2.5 Flash Image to generate fig. 3B. The left image is the original, cropped image, the middle image is an intermediate step, while the right image is the final image used in the manuscript.
